# *Leishmania* HASP and SHERP Genes Are Required for *In Vivo* Differentiation, Parasite Transmission and Virulence Attenuation in the Host

**DOI:** 10.1371/journal.ppat.1006130

**Published:** 2017-01-17

**Authors:** Johannes S. P. Doehl, Jovana Sádlová, Hamide Aslan, Kateřina Pružinová, Sonia Metangmo, Jan Votýpka, Shaden Kamhawi, Petr Volf, Deborah F. Smith

**Affiliations:** 1 Centre for Immunology and Infection, Department of Biology, University of York, York, United Kingdom; 2 Department of Parasitology, Faculty of Science, Charles University, Prague, Czech Republic; 3 Vector Molecular Biology Section, Laboratory of Malaria and Vector Research, National Institute of Allergy and Infectious Diseases, National Institutes of Health, Rockville, Maryland, United States of America; Washington University School of Medicine, UNITED STATES

## Abstract

Differentiation of extracellular *Leishmania* promastigotes within their sand fly vector, termed metacyclogenesis, is considered to be essential for parasites to regain mammalian host infectivity. Metacyclogenesis is accompanied by changes in the local parasite environment, including secretion of complex glycoconjugates within the promastigote secretory gel and colonization and degradation of the sand fly stomodeal valve. Deletion of the stage-regulated HASP and SHERP genes on chromosome 23 of *Leishmania major* is known to stall metacyclogenesis in the sand fly but not in *in vitro* culture. Here, parasite mutants deficient in specific genes within the HASP/SHERP chromosomal region have been used to investigate their role in metacyclogenesis, parasite transmission and establishment of infection. Metacyclogenesis was stalled in HASP/SHERP mutants *in vivo* and, although still capable of osmotaxis, these mutants failed to secrete promastigote secretory gel, correlating with a lack of parasite accumulation in the thoracic midgut and failure to colonise the stomodeal valve. These defects prevented parasite transmission to a new mammalian host. Sand fly midgut homogenates modulated parasite behaviour *in vitro*, suggesting a role for molecular interactions between parasite and vector in *Leishmania* development within the sand fly. For the first time, stage-regulated expression of the small HASPA proteins in *Leishmania (Leishmania)* has been demonstrated: HASPA2 is expressed only in extracellular promastigotes and HASPA1 only in intracellular amastigotes. Despite its lack of expression in amastigotes, replacement of HASPA2 into the null locus background delays onset of pathology in BALB/c mice. This HASPA2-dependent effect is reversed by HASPA1 gene addition, suggesting that the HASPAs may have a role in host immunomodulation.

## Author Summary

Millions of people around the world are at risk of infection with single-celled *Leishmania* parasites that cause a wide range of infectious diseases of the immune system, the leishmaniases. There is no effective vaccine for these infections while available drugs have toxic side-effects and resistance is an increasing problem. Human infection occurs through the bite of infected blood-feeding sand flies. *Leishmania* parasites live in the sand fly gut, where they complete a complex series of developmental changes to become infective to mammals. The parasites also modify the insect gut to promote their own transmission. Little is known about the molecular regulation of these processes. Recently, we showed that deletion of a small group of related genes from the parasite prevented completion of its development in the sand fly, suggesting a role for these sequences in transmission to the host. This study clarifies the expression pattern of these genes during parasite development and shows that the observed stalling of development is accompanied by changes in parasite-sand fly gut interactions and a loss of parasite transmission. These target genes also influence disease development in the mammalian host, confirming critical roles for their encoded proteins throughout the parasite life cycle.

## Introduction

Kinetoplastid parasites of the genus *Leishmania* cause a diverse spectrum of mammalian infectious diseases, the leishmaniases, ranging from cutaneous and mucosal pathologies to potentially fatal visceral infections [1]. Endemic human cases have been reported on all continents except Australia and Antarctica [2]. *Leishmania* parasites are transmitted by female phlebotomine sand flies (Diptera: Psychodidae: Phlebotominae) of the genera *Phlebotomus* (Old World) and *Lutzomyia* (New World) [3]. All mammalian-infective *Leishmania* species (*spp*.) belong to two characterized subgenera, *L*. *(Leishmania)* [4] and *L*. *(Viannia)* [5]. Within the sand fly, parasites of both subgenera undergo metacyclogenesis, a series of morphological and functional changes that produce mammalian-infective metacyclic promastigotes. This process can be triggered, at least *in vitro*, by various factors including nutrient depletion, reduction of pH and tetrahydrobiopterin levels [6,7]. As recently described [8], mammalian-resident intracellular amastigotes transform into midgut adapted, proliferative promastigotes post blood meal (PBM) and these in turn produce nectomonads (synonymous with elongated nectomonads [9,10]) that mediate midgut attachment [11]. Nectomonads then transform into proliferative leptomonads (synonymous with short nectomonads [9,10]), which produce promastigote secretory gel (PSG) containing filamentous proteophosphoglycan (fPPG) [12,13]. Finally, leptomonads transform into the mammalian-infective, midgut-detached metacyclics [14] and haptomonads which colonise and degrade the stomodeal valve (SV) [15,16].

While little is known about the molecular regulation of metacyclogenesis, several genes have been identified that are specifically expressed in late stages of the process. The best-characterised of these are found at the *L*. *(L*.*) major* cDNA16 locus on chromosome 23 and are termed the *L*. *(Leishmania)* species-specific HASP (hydrophilic acylated surface protein) and SHERP (small hydrophilic endoplasmic reticulum-associated protein) genes (HASPA1, SHERP1, SHERP2, HASPB and HASPA2; [17,18]). The locus is conserved in other *L*. *(Leishmania)* species but divergent in *L*. *(Viannia)* species [19]. The three HASPs are highly related proteins with identical *N*- and *C*-terminal regions. HASPB, the best characterized of these proteins, is trafficked to and tethered at the parasite cell surface by co-translational *N*-myristoylation and post-translational palmitoylation at its *N*-terminal SH4 domain [20,21]. HASPB contains extensive amino acid repeat domains in its central region and these show inter- and intraspecific variations while bearing some resemblance to peptidoglycan and immunoglobulin-binding domains of several bacterial surface proteins [22–24]. HASPB is specifically expressed in *L*. *(L*.*) major* metacyclics and amastigotes, but is only detectable in amastigotes in *L*. *(L*.*) mexicana* [19,23,25]. The HASPA genes, which do not encode amino acid repeats, have identical 5’ untranslated regions (UTRs) and open reading frames (ORFs) but different 3’UTR sequences. These contribute to distinct mRNA expression patterns: HASPA2 mRNA is expressed early in procyclics and peaks in metacyclics, while HASPA1 mRNA is upregulated in metacyclics and amastigotes [17,24,26]. SHERP, a small membrane associated protein, is expressed predominantly in metacyclic parasites, where it localizes to the cytosolic faces of the endoplasmic reticulum and mitochondrion [27] and can fold in the presence of membrane phospholipids, supportive of a role in protein-protein interactions [28]. The *in vitro* binding of SHERP to vacuolar H^+^-ATP synthase components involved in subcellular compartment acidification has led to the hypothesis that SHERP may impact on parasite autophagocytosis [28], a process shown to be essential for metacyclogenesis [29].

Genetic deletion of the whole cDNA16 locus in *L*. *(L*.*) major*, by homologous recombination, generated mutants that were stalled in metacyclogenesis within the sand fly, predominately in the nectomonad stage [30]. By contrast, the same mutants showed no significant phenotype when maintained in *in vitro* culture with the culture-generated metacyclics proving more virulent than the parental parasite line (Friedlin V1; FVI) in BALB/c mice [31]. In the same study, episomal-replacement of the full cDNA16 locus into the null background led to unregulated HASP and SHERP overexpression and avirulence [31]. Conversely, more recent reintegration of the whole cDNA16 locus into its former location on chromosome 23 re-established parental line gene regulation and rescued metacyclogenesis *in vivo* [30]. The replacement of an episomal HASPB copy alone into the null background suggested that HASPB was key for the completion of metacyclogenesis *in vivo* [30]. However, since unregulated episomal expression can cause misleading phenotypes in *Leishmania*, verification of this observation by HASPB reintegration into the original cDNA16 locus became essential.

The main focus here was to investigate the contribution of the individual HASPs and SHERP to metacyclogenesis within the sand fly midgut and to host transmission, utilising a broad panel of newly-validated genetic mutants, generated and rigorously tested *in vitro* for this study. While these aims were not fully met, passaging all mutant lines through sand flies to investigate the respective contribution of the HASP and SHERP proteins to metacyclogenesis *in vivo* revealed clear differences in gene expression and parasite behaviour when comparing *in vitro* and *in vivo* conditions. This stimulated a first investigation into the potential impact of midgut factors on gene regulation in *Leishmania*. Further experiments with a sub-group of mutants confirmed that completion of metacyclogenesis, PSG formation, SV colonisation and parasite-to-host transmission are dependent on HASP and SHERP genes. Use of these mutants also allowed us, for the first time, to address the previously established differences in HASPA1 and HASPA2 mRNA expression at the protein level and to investigate the respective contribution of HASPA1 and HASPA2 to amastigote virulence *in vivo*.

## Results

To address our understanding of metacyclogenesis and host transmission *in vivo*, we focused firstly on the *in vitro* generation and characterization of new HASP/SHERP replacement mutant lines; secondly, on the *in vivo* infectivity of cultured mutant lines; and finally, on the impact of genes in the cDNA16 locus on parasite development in the sand fly and transmission by sand fly bite.

### Generation and characterisation of new L. (L.) major HASP and SHERP gene replacement lines

For this study, a total of seventeen new *L*. *(L*.*) major* HASPA1, SHERP, HASPB and/or HASPA2 replacement lines were generated by homologous recombination of newly synthesized gene replacement constructs (S1 Fig) into the original chromosomal location of the cDNA16 locus within the null background of the previously characterized cDNA16 double deletion mutant (cDNA16 dKO [31]; Tables 1 and S1). The alternative approach of targeted gene disruption/deletion of individual genes was not possible technically due to high levels of sequence identity and repetition within the cDNA16 locus. For clarity, and due to strong similarities in mutant phenotypes, the data for only 6 representative gene-replacement lines are shown and discussed here (HASPB sKI, SHERP sKI, HASPA1 sKI, HASPA2 sKI, HASPA1/2 sKI & HA1/2+S2/HB sKI; Table 1). Additional data from other lines and clones can be viewed in the supplementary files. *L*. *(L*.*) major* FVI (the wild type parental line) and the previously characterized cDNA16 dKO and full cDNA16 locus single replacement mutant lines (cDNA16 sKI [30]) served as controls in all experiments.

**Table 1:** *Leishmania (Leishmania) major* mutant lines.

Line	Clones used	Genotype
MHOM/IL/81/Friedlin/VI (FVI)		+/+
cDNA16 dKO	Original	*ΔcDNA16*::*HYG/ΔcDNA16*::*PAC*
cDNA16 sKI	Original	*ΔcDNA16*::*HYG/ΔcDNA16*::*PAC/ΔPAC*::*cDNA16+NEO*
HASPB sKI	98 (99)	*ΔcDNA16*::*HYG/ΔcDNA16*::*PAC/ΔHYG*::*HASPB+NEO*
SHERP sKI	34 (33)	*ΔcDNA16*::*HYG/ΔcDNA16*::*PAC*^*/*^*ΔHYG*::*SHERP2+NEO*
HASPA1 sKI	3 (8)	*ΔcDNA16*::*HYG/ΔcDNA16*::*PAC/ΔPAC*::*HASPA1+BSD*
HASPA2 sKI	18 (16)	*ΔcDNA16*::*HYG/ΔcDNA16*::*PAC/ΔPAC*::*HASPA2+NEO*
HASPA1/2 sKI	8 (18)	*ΔcDNA16*::*HYG/ΔcDNA16*::*PAC/ΔPAC*::*HASPA1-HASPA2+NEO*
HA2+ S2/HB sKI	11 (12)	*ΔcDNA16*::*HYG/ΔcDNA16*::*PAC/ΔPAC*::*HASPA2+NEO/ΔHYG*:: *SHERP2-HASPB+BSD*
HA1/2+ S2/HB sKI	4 (2)	*ΔcDNA16*::*HYG/ΔcDNA16*::*PAC/ΔPAC*::*HASPA1-HASPA2+NEO/ΔHYG*:: *SHERP2-HASPB+BSD*

HA2 = HASPA2; HA1/2 = HASPA1/2 = HASPA1 & HASPA2; S2/HB = SHERP & HASPB; dKO = double deletion (knock-out); sKI = single replacement (knock-in); cDNA16 = complete cDNA 16 locus. “Clone used” indicates the number used to identify the different clones per mutant line used in this study as seen in S2 and S4–S9 Figs. The number in () indicates the alternative clone used to exclude clonal phenotypes and to complement data from the primary clone.

**Fig 1:**
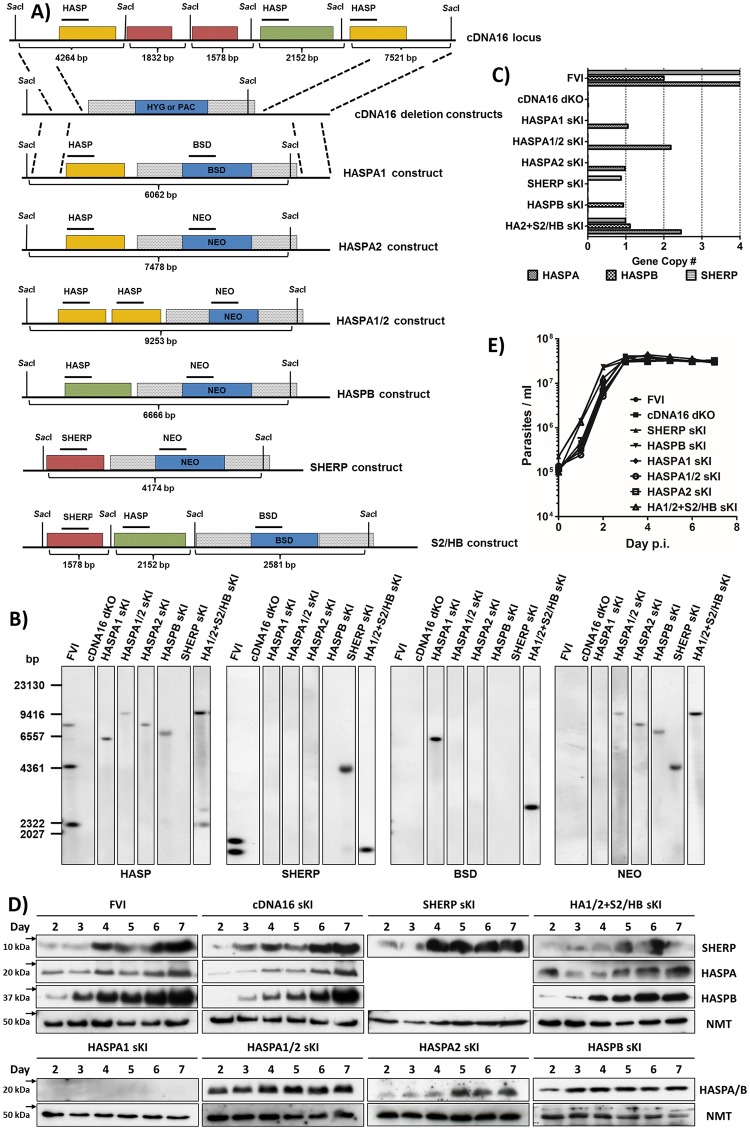
Targeted complementation of HASP and SHERP genes into the cDNA16 locus. A) Schematic representation of the cDNA16 locus and newly synthesised replacement constructs used. Flanking regions targeted for homologous recombination are indicated by dotted lines between top three panels and were the same for all constructs. The top row shows the cDNA16 locus as described by Flinn and Smith (1992); the second row describes the deletion constructs used by McKean et al. [31] for the cDNA16 dKO line generation. Rows 3–8 show the HASP and SHERP replacement constructs used in this study for mutant generation. Refer to S1 & S2 Figs for further details on replacement constructs and constructs integrated into the different mutant lines, respectively. Correct integration of NEO or BSD marker cassettes containing HASP and SHERP replacement constructs into the null background deleted either the HYG (hygromycin resistance gene) [34] or PAC (puromycin resistance gene) [35] marker cassettes in cDNA16 dKO. The solid horizontal bars above the replacement constructs indicate the binding sites of the DIG-labelled Southern blot probes used in (B) below. SacI restriction at the sites shown generates the fragment sizes indicated below each construct, as detected in (B). B) Southern blots of SacI-digested gDNA from the selected mutant line clones (HASPA1 sKI (3), HASPA1/2 sKI (18), HASPA2 sKI (8), HASPB sKI (98), SHERP sKI (34), HA1/2+S2/HB sKI (4)) and the FVI and cDNA16 dKO controls. Data for the same clones are shown in the following figures unless indicated otherwise. The probes used on Southern blots are indicated in (A). Refer to S4 Fig for full set of blots.C) Quantitative PCR of gDNA from culture-derived parental (FVI) and mutant lines shown in (B) to determine construct copy number integration. For single copy integration, a value of 1(±0.2) was predicted, with the exception of the HASPA1/2 construct, containing two HASPA copies with identical ORFs, where a value of 2(±0.5) was expected. Refer to S5 Fig for complete analysis of tested clones.D) Immunoblot analysis of HASPA, HASPB and SHERP expression in culture-derived parental (FVI) and mutant lines (as in A) over a 7 day time course. The blots demonstrate stage specific expression of HASP and SHERP in the mutant lines; NMT is present as a loading control. For more details, refer to S6 & S7 Figs.E) Growth assay in M199 culture of the parental (FVI) and mutant lines shown in (A). No significant growth defects due to genetic manipulation were observed in any of the mutant lines. Refer to S8 Fig for complete analysis of tested clones.

Briefly, newly generated HASP and/or SHERP replacement lines were rigorously tested *in vitro* to ensure correct construct integration and regulated expression in the former cDNA16 locus, using a series of standard protocols, and selected clones (at least two per genotype) were then used for further analyses. Clones were initially screened by PCR (S2 Fig), followed by Southern blot and qPCR analysis to ensure correct integration of HASP and SHERP gene replacement constructs (Figs 1A, 1B, 1C and S2–S5). For Southern blot analysis, genomic DNA (gDNA) from selected clones was *Sac*I digested, size separated and probed with suitable DIG-labelled DNA fragments (Figs 1B and S4). The HASP probe hybridized to HASPA2, HASPA1 and HASPB in FVI, detecting the previously observed 7.6 Kb, 4.3 Kb and 2.2 Kb fragments, respectively [30]. The HASPA1 sKI, HASPA1/2 sKI, HASPA2 sKI and HASPB sKI mutants also showed single fragments of the expected sizes (6.1 Kb, 9.3 Kb, 7.5 Kb and 6.7 Kb, respectively), while no fragments were observed in the cDNA16 dKO and SHERP sKI mutant lines. The HA1/2+S2/HB sKI mutant line, containing all the cDNA16 locus gene types, showed two expected fragments: one of 9.3 Kb equivalent to that detected in the HASPA1/2 sKI mutant line (due to integration of the same HASPA1/2 construct); the second of 2.2 Kb, matching the HASPB fragment detected in FVI, as expected. The SHERP probe confirmed the previously observed 1.8 Kb and 1.6 Kb fragments in FVI [30] and hybridized to an expected 4.2 Kb fragment in SHERP sKI and to an expected 1.6 Kb fragment in HA1/2+S2/HB sKI, matching one of the SHERP fragments in FVI. The BSD (blasticidin resistance) gene [32] was only detected in the HASPA1 and SHERP/HASPB (S2/HB) constructs; a single fragment each in the HASPA1 sKI (6.1Kb) and in the HA1/2+S2/HB sKI (2.6 Kb) mutant lines as expected, with no BSD hybridising fragments in FVI and cDNA16 dKO. The NEO (neomycin resistance) gene [33], present in the HASPA1/HASPA2 (HASPA1/2 or HA1/2), HASPA2, HASPB and SHERP constructs, generated fragments of the same size as those detected by the HASP and SHERP probes since there was no *Sac*I restriction site between the gene of interest and the resistance marker cassette (Figs 1A and S3). The Southern analysis also excluded the presence of multi-copy episomal constructs in the parasite lines.

Gene copy number in selected clones was verified by gene-specific qPCR (Figs 1C and S5). The Na/H antiporter-like protein gene, present as a single copy on chromosome 23, was used as control for data normalization. A value of 1(±0.2) was predicted for all targets (HASPA1, SHERP, HASPB and HASPA2 gene constructs) present as single gene copies. Only HASPA1/2 sKI and HA1/2+S2/HB sKI, which contained both HASPA1 and HASPA2 in the HASPA1/2 (HA1/2) construct were expected to generate a value of 2(±0.5) for HASPA. As shown in Fig 1C (see also S5 Fig), the expected values were obtained for each target gene within the mutant lines.

Two to three selected clones from each line were then passaged through BALB/c mice to restore parasite infectivity and stable, regulated gene construct expression (which is known to diminish for the HASP and SHERP genes following prolonged parasite passage in culture [21]). Immunoblotting was used to verify stage-specific protein expression from the integrated HASP and SHERP genes, using whole lysates generated from low-passage parasites cultured over a 6 day time course from day 2 to day 7 post inoculum (p.i.), in comparison with HASPB, HASPA and SHERP expression patterns in FVI (Fig 1D). While only one clone per line is shown here, additional clones showed comparable results for each parasite line, respectively (S6 Fig), as demonstrated in S7 Fig for HASPA2 sKI and HASPA1/2 sKI. Due to its small molecular mass and biophysical properties, SHERP is inherently difficult to transfer onto nitrocellulose, reducing blot quality. However, the results for HASPB and SHERP showed that integration of the HASPB and SHERP constructs into the cDNA16 locus was sufficient to reproduce the parental line (FVI) protein expression patterns. The unexpected differences in HASPA expression between HASPA1 sKI, HASPA1/2 sKI and HASPA2 sKI mutant lines are discussed further below. The constitutively expressed *L*. *(L*.*) major N*-myristoyltransferase protein (NMT; [36]) served as a loading control in all protein analyses.

Selected mutants were further tested for any growth defects due to genetic manipulation. Low-passage parasites were inoculated at 10^5^ parasites/ml into M199 medium and cell numbers monitored every 24 hr for 7 days. This growth assay was performed in 3–4 consecutive repeats, inoculating fresh M199 medium to a final concentration of 10^5^ parasites/ml using the previous cultures at late log-phase (day 3 p.i.). No significant fitness defect was observed in any of the mutant lines growing as *in vitro* promastigotes in comparison to the parental line (FVI; Figs 1E and S8). All selected clones reached stationary phase by day 3–4 p.i., correlating with peak parasite density, followed by a similar rate of decline in live parasite density to day 7 p.i. with no statistically significant differences.

### Differential stage specific expression of the HASPA1 and HASPA2 proteins

Since HASPA1 and HASPA2 have identical ORFs, it is impossible to distinguish these two proteins by antibody probing in wild type parasites [26]. Generation of individual HASPA1 and HASPA2 replacement mutant lines in this study allowed analysis of protein expression from these two genes, previously only possible at the mRNA level [17,18]. The immunoblot data from promastigotes (Figs 1D, S6 and S7) showed a similar HASPA expression in HASPA2 sKI as in FVI and cDNA16 sKI, peaking at day 7 p.i. when cultures were enriched in metacyclics. This correlated with the previously established HASPA2 mRNA expression pattern in FVI [17,18], although the correlation was less clear for HASPA2-only combination mutants with HASPB and/or SHERP (S6 Fig). In contrast, HASPA protein was not detectable in HASPA1 sKI and in HASPA1-only combination mutants cultured up to 7 days (Figs 1D and S6) regardless of the clone tested, while low level mRNA expression had previously been detected in metacyclic cells [17,18].

**Fig 2:**
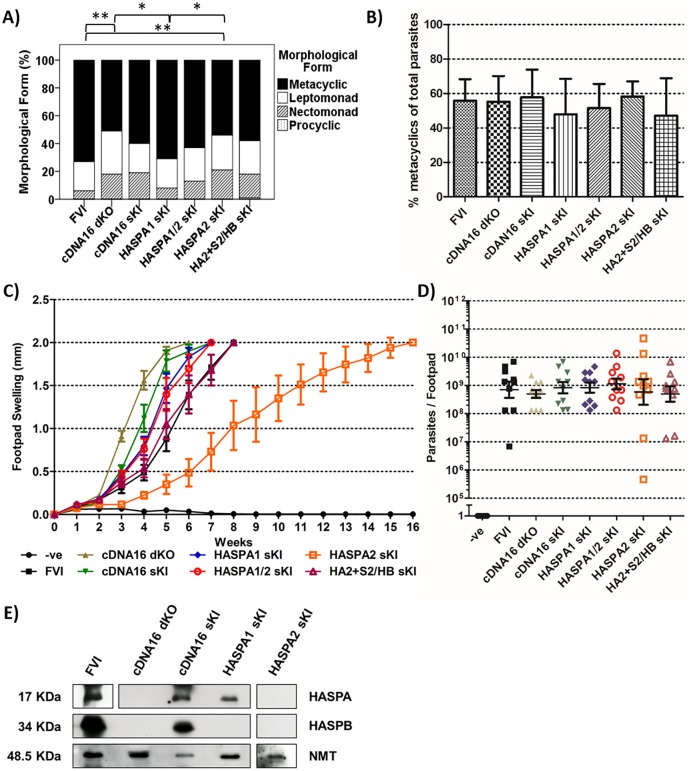
Infection Assays. A) Parasite morphology of cloned lines. Wild type and mutant promastigotes generated by amastigote inoculation into M199 culture at 26°C were harvested at day 7 p.i., stained, measured and classified as procyclics, nectomonads, leptomonads and metacyclics, according to Sádlová et al. [30] (as adapted from Walters [37] and Ciháková and Volf [10]). n = 100 per bar; differences between bars were analysed by χ^2^-square test with P≤0.05 considered as significant and represented by asterisks (* = P≤0.05 and ** = P≤0.01). B) Agglutination assay. Late-stage parasite cultures (day 7 p.i.) were agglutinated with peanut lectin; non-agglutinated parasites are defined as metacyclics. The bar graph shows metacyclic content per strain in late-stage culture as percentage of total parasites pre-agglutination. C) Lesion development in BALB/c mice. The experiment was conducted with two distinct clones per tested L. (L.) major lines to exclude clonal phenotypes. Five mice were infected with 10^7^ parasites per parental line (FVI) and the cDNA16 dKO, cDNA16 sKI, HASPA1 sKI (3 or 8), HASPA1/2 sKI (18 or 16), HASPA2 sKI (18 or 8) or HA2+S2/HB sKI (11 or 12) mutants, all grown to day 6 p.i. in culture, by subcutaneous injection into the right hind footpad. Lesion development was monitored until the lesions reached 2 mm in diameter; the mean lesion diameter is shown ±SE (n = 10). The data generated from each clone are shown separately in S9 Fig. D) The parasite load for each infected footpad from A) was determined in a limiting dilution assay at the final time point for each mouse. Mean parasite burdens per footpad are shown ±SE (n = 10). Parasites were detected in all infections with the exception of the PBS negative control. The data generated from each clone are shown separately in S9 Fig. E) Immunoblot analysis of lysed amastigotes. Whole lysates of amastigotes purified from infected mouse material were separated on 12% SDS-polyacrylamide gels, blotted and probed for HASPA, HASPB and NMT (as a loading control). The HASPA exposure for FVI was over a shorter interval (30 sec) than for the other lines (1 min), as the HASPB signal bled into the HASPA signal at longer exposure. Exposure time for HASPA and HASPB in HASPA2 sKI was 5 min to ensure no weak fragments were overlooked.

Conversely, immunoblots of whole lysates of intracellular amastigotes purified from skin lesions of BALB/c mice showed expression of HASPA in the HASPA1 sKI amastigotes, but no detectable HASPA expression in the HASPA2 sKI amastigotes (Fig 2E). Overall, this analysis has for the first time demonstrated that protein expression from the individual HASPA genes is stage-specific in *L*. *(L*.*) major*, with HASPA1 expressed in amastigotes and HASPA2 in promastigotes.

Interestingly, the presence of both HASPA1 and HASPA2 in the same construct appeared to enhance and deregulate HASPA expression in HASPA1/2 sKI and HA1/2+S2/HB sKI promastigotes (Fig 1D; and in other HASPA1-HASPA2 combination mutants with HASPB or SHERP; S6 Fig) regardless of the clone tested (S7 Fig), suggesting that either HASPA1 contributes to HASPA expression in early culture stages in the presence of HASPA2 or that the HASPA2 gene has lost parental line regulation in the HASPA1/2 construct (Figs 1D and S6). The latter explanation implicates cDNA16 locus structural constraints as important for correct HASPA regulation. This is a valid hypothesis since the HASPA1/2 construct was assembled with the same DNA fragments as the HASPA1 and HASPA2 constructs (S1 Fig), the sequence was verified post construct assembly and the different HASPA expression patterns in HASPA1 sKI and HASPA2 sKI correlated with the previously established mRNA expression patterns [17,18]. However, this unexpected HASPA1/2 mutant phenotype did not result in an unexpected phenotype *in vivo*.

### Virulence of HASPA mutants in vivo

Our previous work had shown that deletion of the full cDNA16 locus enhanced footpad lesion development in BALB/c mice following inoculation with cDNA16 dKO as compared to FVI promastigotes [31]. Due to our new observations on stage specific expression of HASPA1 and 2 in amastigotes and promastigotes, respectively, we wanted to revisit the original *in vivo* phenotype and address whether deletion and replacement of these genes affected footpad pathology. For that purpose, two clones from each of seven parasite lines (FVI, cDNA16 dKO cDNA16 sKI, HASPA1 sKI, HASPA2 sKI, HASPA1/2 sKI and HA2+S2/HB sKI; refer to S9 Fig for clone ID) were grown *in vitro* to late-stationary phase (day 7 p.i.) and assessed for the presence of metacyclic parasites. Given the lack of metacyclic-specific markers in *L*. *(L*.*) major* other than SHERP and HASPB, the focus of this study, the efficiency of metacyclogenesis *in vitro* was monitored by two complementary approaches: by morphometric analysis of fixed parasites (Figs 2A and S10), in comparison with existing sand fly data, and by metacyclic purification following peanut lectin agglutination (PNA, Fig 2B).

Morphometry showed that all mutant lines had generated metacyclics (Figs 2A and S10) with FVI and HASPA1 sKI being the most efficient (>70%) and cDNA16 dKO and HASPA2 sKI the poorest (<55%). Statistical significant differences occurred only between: FVI and cDNA16 dKO, P = 0.003; FVI and HASPA2 sKI, P = 0.003; HASPA1 sKI and cDNA16 dKO, P = 0.011; HASPA1 sKI and HASPA2 sKI, P = 0.014. However, metacyclics from all cultured lines and clones were similar in size and morphology (S10 Fig), suggesting no difference in metacyclic quality. Leptomonads were present at a similar percentage (~20%) in all cultured parasites tested.

Metacyclic enrichment by PNA is subject to some losses due to passive entrapment of the highly motile parasites in the lectin aggregates. Despite the observed reduction in metacyclic numbers for some lines as compared to morphometry (Fig 2A and 2B), overall the agglutination test did not reveal any significant differences in the capacity of the different parasite lines to undergo metacyclogenesis (Fig 2B), as verified by morphometric analysis (S10 Fig). Comparing three replicates, FVI generated a mean of 55.8%, cDNA16 dKO 55.2%, cDNA16 sKI 57.7%, HASPA1 sKI 47.9%, HASPA1/2 sKI 51.5%, HASPA2 sKI 58.2% and HA2+S2/HB sKI 47.2% metacyclics in day 7 cultures. Based on these findings, the numbers of metacyclic parasites inoculated into the footpads of BALB/c mice were very similar for all clones tested.

Given the demonstrated consistency of metacyclogenesis in these clones, and to allow direct comparison with previous observations, cultured promastigotes were harvested and injected at 3x10^7^ parasites per BALB/c footpad without prior metacyclic purification. Disease progression for two independent clones per mutant line (Figs 2C, S9a and S9c) was tested by weekly footpad measurements followed by limiting dilution assay (LDA) of footpad homogenates once ~2 mm lesion diameter was reached (Figs 2D, S9b and S9d). This analysis showed that both cDNA16 dKO clones were fast to develop severe lesions (within 5 weeks p.i.), as previously observed [31], although this was not due to elevated numbers of parasites within the lesions (Figs 2C, 2D and S9). The parental line (FVI) clones required ~8 weeks p.i. to reach the same level of lesion development, while cDNA16 sKI required ~6–7 weeks. Interestingly, both HASPA2 sKI clones showed a delayed onset of footpad lesion development compared to FVI, which was also repeatedly observed during routine passaging of parasites through BALB/c mice, using these two distinct clones. HASPA2 sKI-infected BALB/c mice took significantly longer (>10 weeks p.i.; P<0.001) to produce comparable footpad lesions to those observed in the other mutants. Conversely, lesion development in HASPA1 sKI and HASPA1/2 sKI infected footpads had a similar time course to cDNA16 sKI (~6–7 weeks). Lesion development in HA2+S2/HB sKI was comparable to FVI, suggesting that addition of a HASPB and/or SHERP copy restored FVI virulence levels. Further investigation will be required to resolve the mechanisms whereby the HASPs and SHERP contribute to disease outcome.

### Development of HASP and SHERP replacement mutant lines in the sand fly vector

**Fig 3:**
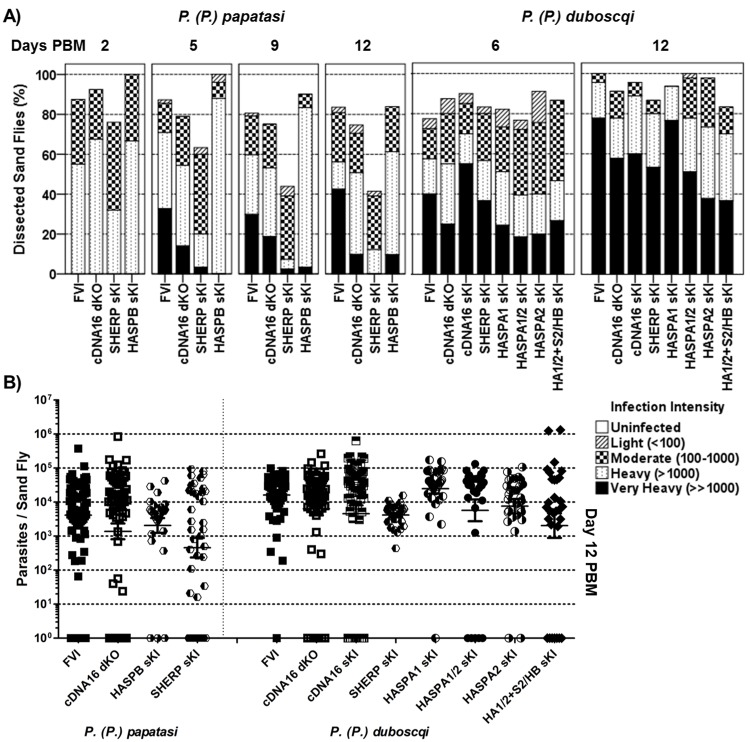
Analysis of parasite infection intensities in the sand fly midgut. A) Rates and intensity of infections with representative parasite lines were scored by light microscopy at day 2, 5 or 6, 9 and 12 PBM. Infections were considered according to infected sand fly species and scored as light (<100 parasites/gut), moderate (100–1000 parasites/gut), heavy (>1000 parasites/gut) and very heavy (>>1000 parasites/gut) as described in Materials and Methods. Sand fly infection experiments were repeated at least 3x for each line. Differences between lines were analysed by χ^2^ test: in P. (P.) papatasi day 2 PBM, P = 0.083, χ^2^ = 11.18, df = 6; day 5 PBM, P<0.001, χ^2^ = 55.7, df = 12; day 9 PBM, P<0.001, χ^2^ = 72.05, df = 12; day 12 PBM, P<0.001, χ^2^ = 74.422, df = 12; and in P. (P.) duboscqi day 6 PBM, P = 0.028, χ^2^ = 38.835, df = 24; and day 12 PBM, P<0.001, χ^2^ = 55.95, df = 24. B) Light microscopic scoring was verified by qPCR analysis of parasite DNA content per midgut of 30 sand flies at day 12 PBM per parasite line shown in A). Data were normalized (log_10_[x+1] transformation) and analysed by a non-parametric one-way ANOVA (Kruskal-Wallis test + Dunns pair wise post-test): in P. (P.) papatasi no significant difference was described (P = 0.1615); the same test showed a significant difference for tested L. (L.) major lines in P. (P.) duboscqi (P<0.001). A Dunn’s Multiple Comparison post-test showed significant difference in HASPB sKI vs. FVI, cDNA16 dKO, cDNA16 sKI, HASPA1 sKI, HASPA1/2 sKI (all P≤0.001) and HASPAs sKI (P≤0.05); SHERP sKI vs. FVI (P≤0.01), cDNA16 sKI (P≤0.05), HASPA1 sKI (P≤0.001) and HASPA1/2 sKI (P≤0.05), HASPA1 sKI vs. HASPA1/2 sKI (P≤0.05).

**Fig 4:**
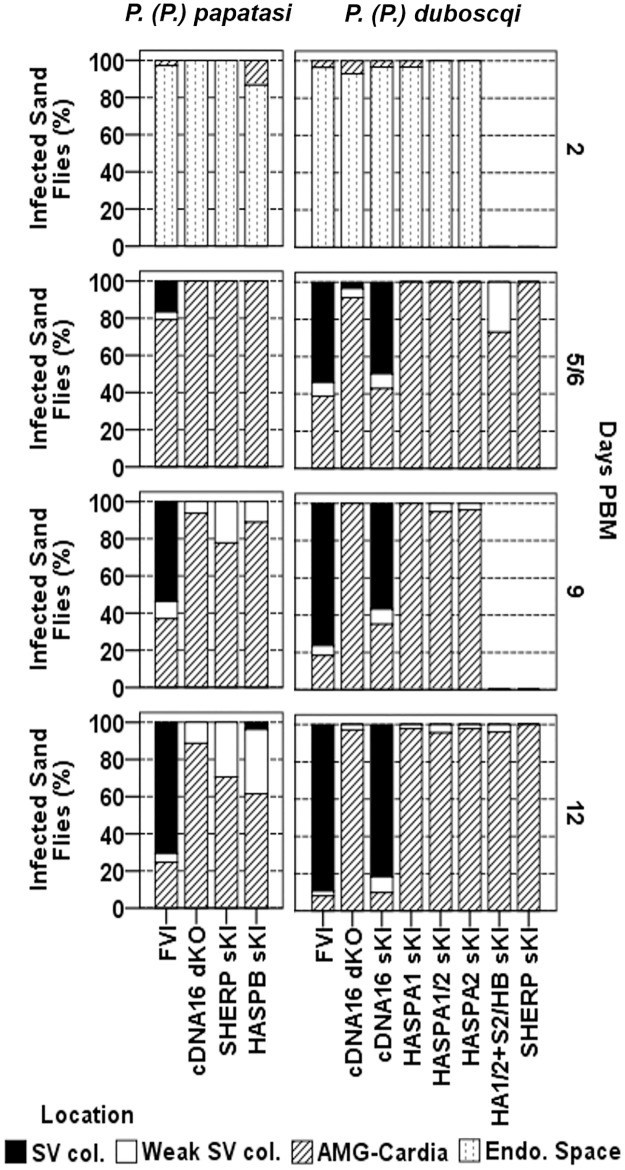
Parasite localization in the sand fly midgut over time. In addition to the infection intensity scoring in Fig 3, parasite localization was also recorded. Freshly dissected intact midguts were analysed by light microscopy and scored (as adapted from Sádlová et al. [30]) according to localization: encapsulated in the peritrophic matrix (Endo. Space), in the midgut lumen of the abdominal midgut (AMG) and thoracic midgut (TMG) (AMG-Cardia), weak colonization of the stomodeal valve (SV) (Weak SV col.) and strong, parental like SV colonization (SV col.). Differences between lines were analysed by χ^2^ test: in P. (P.) papatasi day 2 PBM, P = 0.054, χ^2^ = 7.648, df = 3; day 5 PBM, P = 0.003, χ^2^ = 20.002, df = 6; day 9 PBM, P<0.001, χ^2^ = 67.065, df = 6; day 12 PBM, P<0.001, χ^2^ = 99.336, df = 6; and in P. (P.) duboscqi day 6 PBM, P<0.001, χ^2^ = 154.455, df = 12; and day 12 PBM, P<0.001, χ^2^ = 308.988, df = 12.

**Fig 5:**
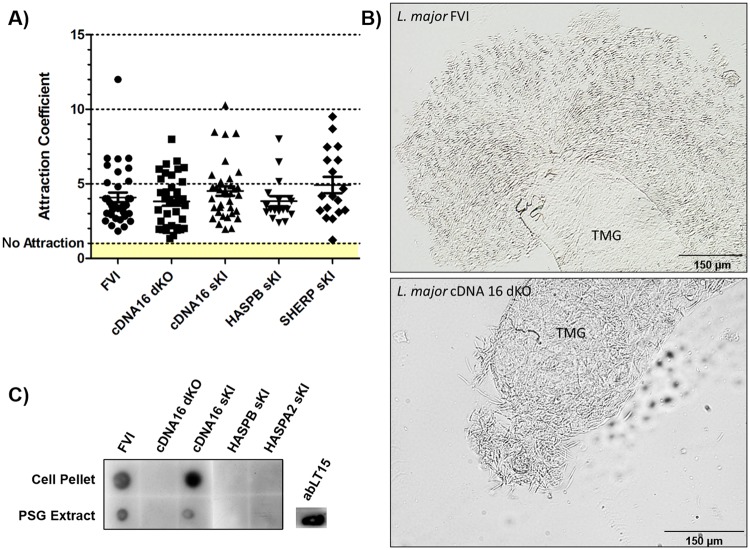
Osmotaxis and PSG detection assays. A) The osmotaxis assays analysed the capacity of each parasite line tested to follow attractant gradients in vitro. Attractant coefficients were calculated (dividing parasite numbers attracted by sucrose by parasite numbers in attractant free capillaries). No significant differences in comparison to the parental line (FVI) were shown by parametric one-way ANOVA (P = 0.206). B) Light microscopic images of compressed infected thoracic midguts (TMG) dissected from FVI and cDNA16 dKO infections. Parasites immobilized in PSG spread around the ruptured stomodeal valve (SV), allowing visualisation of the PSG, as shown for FVI. In TMGs lacking PSG, this is not observed on squeezing the midgut, as shown for cDNA16dKO. The images were marginally adjusted for contrast and sharpness to improve visibility. C) PSG extracts from 10 infected midguts per parasite line were dot-blotted on activated nitrocellulose membranes and probed with the LT15 antibody. PSG was only detected in FVI and cDNA16 sKI. LT15 was blotted onto the nitrocellulose membrane as a control for detection by the appropriate secondary HRP antibody.

Sádlová *et al*. [30] showed that stalling of metacyclogenesis due to cDNA16 locus deletion is only observed in the sand fly vector and not in culture. In this new study, each of the *L*. *(L*.*) major* mutant lines (Tables 1 and S1) was fed independently at 10^6^ early log-phase promastigotes/ml blood to *P*. *(P*.*) papatasi* and/or *P*. *(P*.*) duboscqi*, both *L*. *(L*.*) major*-specific vector species [8]. A total of 2,736 sand flies (S2 Table) were sampled at different time points (day 2, 5, 9 and 12 PBM or at day 6 and 12 PBM only) and infection loads, parasite localization and parasite morphology assessed (Figs 3–5 and S11–S14).

Infection load data by microscopy (Figs 3A and S11) revealed that *P*. *(P*.*) duboscqi* supports significantly higher (P<0.001) parasite numbers in the midgut than *P*. *(P*.*) papatasi*. In *P*. *(P*.*) papatasi*, significant differences in parasite load were observed between FVI and the three mutant lines tested, cDNA16 dKO, HASPB sKI and SHERP sKI, after blood meal defecation (day 5, 9 and 12 PBM; P< 0.001; Figs 3A and S11). While FVI, cDNA16 dKO and HASPB sKI showed significantly increased parasite loads from day 2 PBM to day 12 PBM (P<0,001; P = 0.009; P = 0.032, respectively), SHERP sKI survived comparatively poorly (~40% infected at day 12 PBM) with significantly decreased persistence of infection from day 2 PBM to day 12 PBM (P = 0.027). Interestingly, SHERP sKI survival in *P*. *(P*.*) duboscqi* was not affected (~85% at day 12 PBM) and developed as well as other mutant lines tested (Figs 3A and S11). In general, parasite lines survived well in *P*. *(P*.*) duboscqi*, showing significant increases in parasite numbers (P≤0.002) from day 6 PBM to day 12 PBM (S11 Fig). The microscopically evaluated parasite loads were verified for day 12 PBM samples by qPCR for 30 infected female sand flies per parasite line (Figs 3B and S12). Although microscopy tended to underestimate infection loads compared to qPCR, there was generally good correlation between light microscopic and qPCR data with the exception of cDNA16 dKO and SHERP sKI in *P*. *(P*.*) papatasi*, which showed significantly higher parasite loads by qPCR than by microscopy (Figs 3B and S12). However, while microscopic analysis only evaluates live parasites, qPCR does not discriminate between live and dead parasites containing genomic DNA. No significant differences were established between parasite lines tested in *P*. *(P*.*) duboscqi* at day 12 PBM by qPCR with the exception of FVI and HASPA1 sKI compared to HASPA2 sKI (P = 0.01 and 0.004, respectively) and HA1/2+S2/HB sKI (P = 0.03 and 0.01, respectively).

In parallel, parasite localization was assessed during the course of sand fly infection. Parasites were generally observed in the endoperitrophic space by day 2 PBM and within the midgut lumen by day 5/6 PBM after blood meal defecation (Figs 4, S13 & S3–S5 Tables). On rare occasions, blood meal remnants were present in the AMG and hindgut by day 5/6 PBM. Invasion of the TMG (thoracic midgut) was observed to varying degrees by day 5/6 PBM and this increased in frequency and intensity by day 9 and 12 PBM (Figs 4, S13 & S3–S5 Tables). Infections with FVI and cDNA16 sKI concentrated strongly in the TMG by day 9 and 12 PBM and these were accompanied by TMG distention. All other mutant lines tested did not show this distension effect, with parasites being either evenly spread from the cardia to the posterior of the AMG or largely found in the AMG by day 12 PBM. An *in vitro* assay was used to demonstrate that parasite osmotaxis was not significantly compromised in any of the mutant lines compared to FVI; this could, therefore, be excluded as a factor affecting parasite accumulation in the TMG (Fig 5A). The observed TMG distention in late stage infection in FVI and cDNA16 sKI parasites only was indicative of the presence of PSG, an attachment matrix for leptomonads and nectomonads but proposed to be traversable by metacyclics (communication by Matthew E. Rogers). Mutant line infections lacked this gel, as observed by light microscopy (Fig 5B), suggesting compromised PSG generation in the mutant lines *in vivo*. To further investigate this observation, PSG was extracted from infected sand flies by pooling 10 infected midguts per infecting line. Samples were dot-blotted to activated nitrocellulose membranes and probed with the LT15 antibody that recognises *L*. *major* fPPGs, major components of the PSG [38]. PSG was only detected in FVI and cDNA16 sKI, but was undetectable in the other mutants tested. While parasites from all tested lines reached the cardia, significant differences were observed in the efficiency of SV colonization. FVI and cDNA16 sKI were the only lines tested to efficiently colonize the SV (Figs 4, 5B and 5C). While SHERP sKI and HASPB sKI were observed to attach at low numbers to the SV in *P*. *(P*.*) papatasi* (29.4% and 38.4% of analysed infected sand flies, respectively), mutant lines infecting *P*. *(P*.*) duboscqi* colonized the SV only weakly (<5% of cases; Fig 4), probably due to reduced haptomonad generation, the only parasite forms that attach to the SV. This hypothesis could not be verified due to the lack of any haptomonad specific markers.

**Fig 6:**
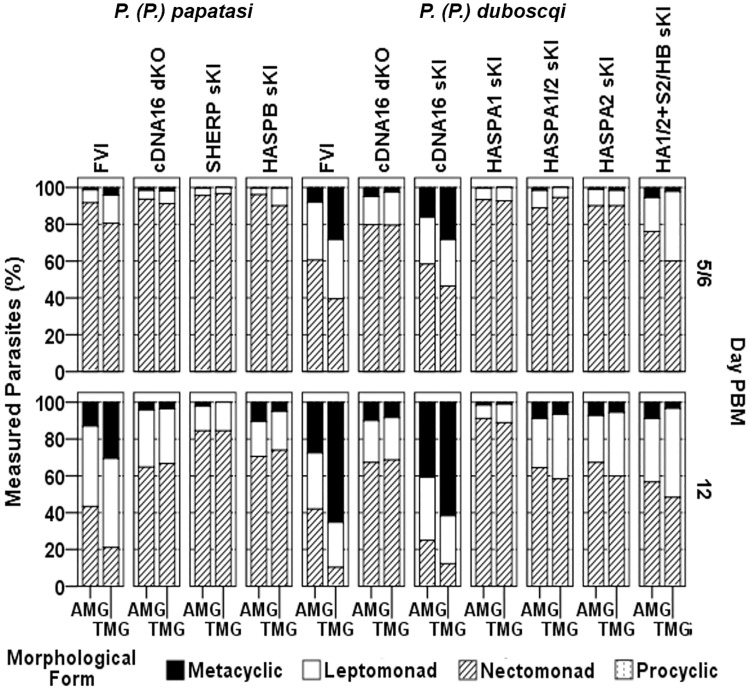
Morphology of sand fly midgut-derived promastigotes. Promastigotes from midguts analysed in Figs 3 & 4, collected at day 5 or 6, 9 and 12 PBM, were classified by morphology as procyclics, nectomonads, leptomonads and metacyclics according to Sádlová *et al*. [30]; adapted from Walters [37] and Ciháková and Volf [10]. The frequencies (%) of each morphological form are shown for each parasite line, analysed according to the infected sand fly species. Differences between lines were analysed by *χ*^2^ test: in *P*. *(P*.*) papatasi* day 5 PBM, P = 0.001, *χ*^2^ = 22.27, df = 6; day 9 PBM, P<0.001, *χ*^2^ = 84.457, df = 6; day 12 PBM, P<0.001, *χ*^2^ = 254.494, df = 6; and in *P*. *(P*.*) duboscqi* day 6 PBM, P<0.001, *χ*^2^ = 143.975, df = 12; and day 12 PBM, P<0.001, *χ*^2^ = 630.013, df = 12.

Parasite morphology was analysed on Giemsa-stained gut smears from day 5/6, 9 and 12 PBM, using measurements of flagellum length, cell body length and width with separate analysis in AMG and TMG. This analysis showed significant differences in midgut metacyclogenesis between *L*. *(L*.*) major* lines at different time points (Figs 6 and S14). Only FVI and cDNA16 sKI produced metacyclics efficiently by day 12 PBM (P<0.001), compared to all other lines tested in the same vector species, although FVI metacyclic generation was significantly more efficient in *P*. *(P*.*) duboscqi* by day 12 PBM than in *P*. *(P*.*) papatasi* (P<0.001). These observations confirm that all other mutant lines tested could not complete metacyclogenesis *in vivo*, although they did so *in vitro* (Fig 2A and 2B). In addition, metacyclics were preferentially present in the TMG, while leptomonads were equally represented in the AMG and TMG (Figs 6 and S14) and nectomonads were preferentially found in the AMG. Thus all mutant lines, except cDNA16 sKI, resembled the cDNA16 null background with very few metacyclic-like parasites present at day 12 PBM and no gradient of differentiated parasites towards the TMG, correlating with the lack of parasite accumulation in the TMG and PSG-deficiency in these lines. Leptomonad generation in *P*. *(P*.*) duboscqi* showed no significant differences at day 12 PBM between all tested lines, except for HASPA1 sKI, which generated leptomonads very inefficiently (P<0.001, compared to all other lines tested in the same vector species). In *P*. *(P*.*) papatasi*, differences in leptomonad generation were only observed in SHERP sKI (P<0.001, compared to all other lines tested in the same vector species), which produced leptomonads as inefficiently as HASPA1 sKI in *P*. *(P*.*) duboscqi*. Differences in leptomonad generation between FVI and all other mutant lines by day 12 PBM were more pronounced in *P*. *(P*.*) papatasi*. Overall, no mutant line tested rescued the full parental line (FVI) phenotype, with the exception of cDNA16 sKI.

### Differences in gene expression in the mutant lines tested in vivo or in vitro

**Fig 7:**
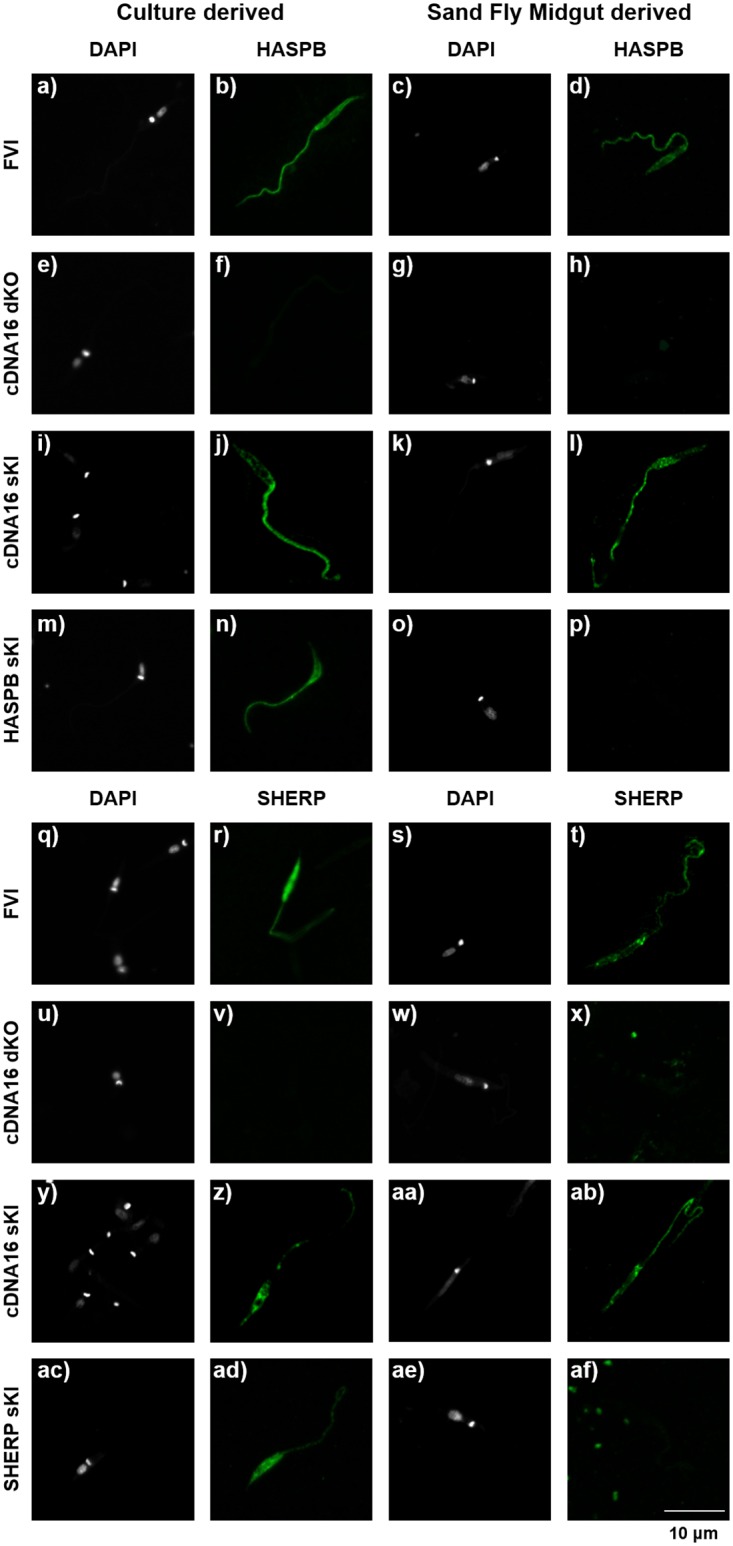
Analysis of cultured and midgut-derived metacyclic parasites by confocal imaging. Detection of HASPB and SHERP expression by immunofluorescent antibody labelling in the parental line (FVI), cDNA16 sKI, cDNA16 dKO, HASPB sKI and SHERP sKI mutants, collected from day 7 p.i. M199 culture and day 12 PBM midguts. DAPI (white) stained nucleus and kinetoplastid DNA; HASPB or SHERP (green) were detected by specific mononuclear antibodies (rabbit), which was tagged by a secondary 488 anti-rabbit antibody. Size bar = 10 μm.

To further characterize HASPB and SHERP expression in the newly generated mutant lines, parasites derived either from culture or from sand fly midguts were fixed, antibody labelled for HASPB or SHERP and analysed by confocal microscopy. Both proteins were clearly detected in metacyclics derived from cultured FVI, cDNA16 sKI, HASPB sKI and SHERP sKI, respectively, while cDNA16 dKO promastigotes did not show fluorescence, as expected (Fig 7). FVI and cDNA16 sKI recovered from sand fly midguts were positive for HASPB and SHERP expression, too. However, unlike *in vitro*, HASPB sKI and SHERP sKI from sand fly midguts produced no detectable HASPB or SHERP signal, respectively. These observations suggest that either the gene regulation observed in these mutants *in vitro* is not replicated *in vivo* or that parasite differentiation is compromised *in vivo*, leading to a loss of HASPB and SHERP expression.

**Fig 8:**
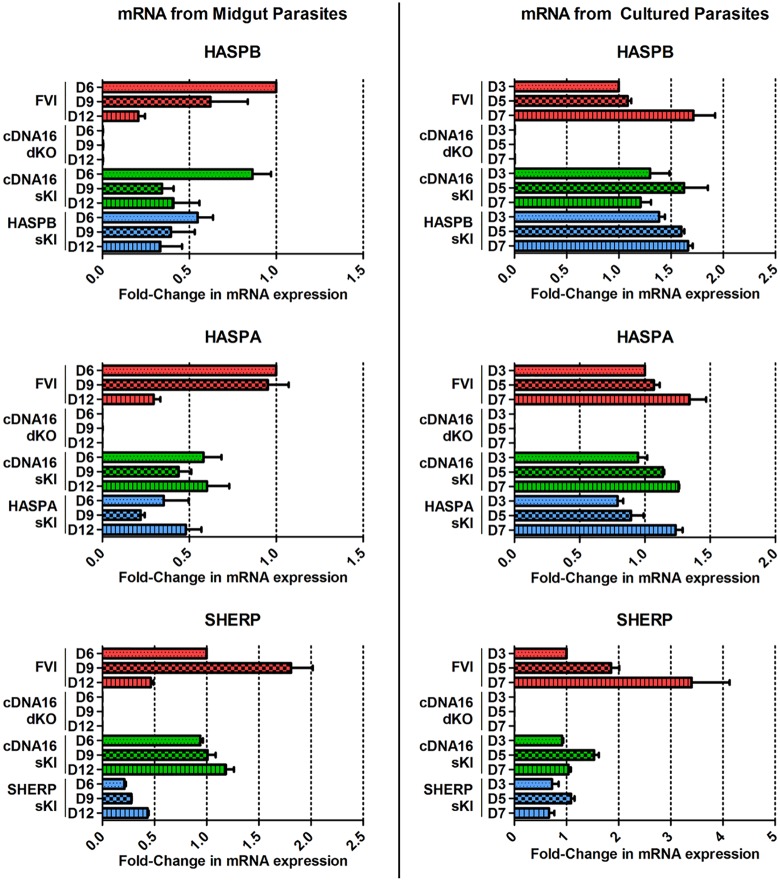
Quantitative PCR analysis of mRNA from cultured and midgut-derived parasites. mRNA was extracted from cultured and midgut-derived FVI, cDNA16 dKO, cDNA16 sKI, HASPAB sKI, SHERP sKI and HASPA2 sKI parasites at day 3, 5 and 7 p.i. and day 6, 9 and 12 PBM, respectively. Mean C_t_ values were normalized against the housekeeping gene NMT and ΔΔC_t_ values were generated against FVI day 6 PBM for midgut-derived and day 3 p.i. for culture-derived samples. Fold-differences for HASPAs, HASPB and SHERP mRNA were plotted.

To investigate whether the lack of HASPB and SHERP expression was due to altered regulation at the protein or mRNA level, qRT-PCR was performed on midgut and culture-derived parasite mRNA. The HASPB mRNA levels of FVI and cDNA16 sKI, both having completed metacyclogenesis *in vivo*, showed elevated HASPB mRNA levels at day 6 PBM *in vivo*, subsequently declining towards day 12 PBM (Fig 8). Interestingly, HASPB sKI from midguts, which had not produced a detectable fluorescent HASPB signal (Fig 7), showed the same expression pattern, although total mRNA levels were lower than in FVI at all time-points, despite statistical compensation for the two HASPB copies in FVI compared to the one copy in HASPB sKI. In culture derived parasites, FVI and HASPB sKI showed similar expression patterns with peak expression of HASPB mRNA at day 7 p.i., although initial mRNA levels were higher (~1.39-fold) at day 3 p.i. in HASPB sKI compared to FVI. HASPB mRNA expression in cDNA16 sKI had already peaked at day 5 p.i. and declined by day 7 p.i. The downregulation of HASPB mRNA *in vivo* towards day 12 PBM was unexpected, since HASPB is expressed in metacyclics and amastigotes, and contradicts the *in vitro* observed mRNA expression pattern, which peaked at the final time-point (day 7 p.i.), when metacyclics were at their densest (Fig 8). For HASPA mRNA analysis, the HASPA2 sKI mutant was used, given that HASPA2 is known to be upregulated in early log-phase growth [18]. In the case of HASPA, the expression patterns of cDNA16 sKI and HASPA2 sKI were comparable to FVI *in vitro*, but distinct from FVI *in vivo*, while similar between cDNA16 sKI and HASPA2 sKI (Fig 8). In the case of SHERP, cDNA16 sKI and SHERP sKI had similar mRNA expression patterns both *in vitro* and *in vivo*, but these were distinct from FVI in both conditions (Fig 8). Overall, these results suggest that dysregulation of gene expression is not responsible for the lack of HASPB and SHERP detection in sand fly derived parasites, since the single-replacement lines either resembled FVI and/or cDNA16 sKI, which both develop normally *in vivo*.

Since it has previously been reported that culture conditions can influence promastigote development [39], we investigated whether the 20% FCS supplemented M199 medium used for *in vitro* culture influenced HASPB and SHERP expression, as compared to growth in 5% sucrose, mimicking the sugar-rich plant sap that provides nutrients in the sand fly midgut after blood meal defecation *in vivo*. Parasites grown for 2 days in M199 were washed and suspended in 5% sucrose/PBS solution, prior to collection of protein samples every 24 hours of the growth cycle. Comparative immunoblots of the whole parasite lysates (S15 Fig) provided no evidence for downregulation of HASPB and SHERP in HASPB sKI and SHERP sKI, respectively, when grown in 5% sucrose conditions, thereby excluding culture conditions as the source of differential HASPB and SHERP expression.

**Fig 9:**
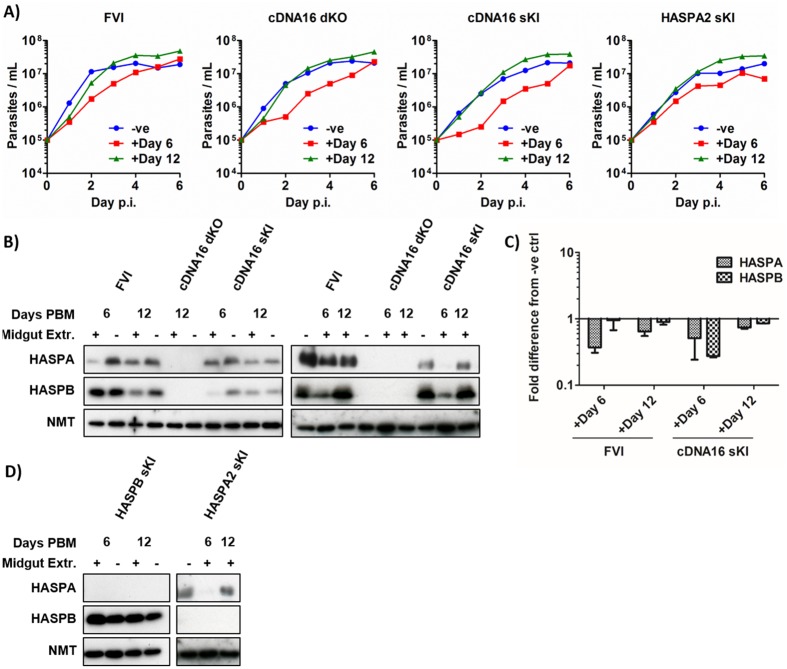
Assessing the impact of different growth conditions on HASP and SHERP expression. A) Growth curves of parasite lines (FVI, cDNA16 dKO, cDNA16 sKI, HASPA2 sKI) without (-ve) or with day 6 (+Day 6) or day 12 (+Day 12) PBM filter-sterilized midgut homogenates in M199 medium. B) Immunoblots from two repeats of FVI, cDNA16 dKO, cDNA16 sKI grown in M199 with (+) or without (-) day 6 and 12 PBM midgut homogenates from uninfected blood-fed female sand flies. C) Fold difference of HASPA and HASPB expression in FVI and cDNA16 sKI grown with day 6 or 12 PBM midgut homogenates compared to no homogenates. cDNA16 dKO served as a negative control for HASPA and HASPB detection. D) Immunoblots of HASPA2 sKI and HASPB sKI grown with or without day 6 or 12 PBM midgut homogenates probed for HASPA and HASPB. NMT served in all blots as a loading control.

An alternative explanation for the observed differences in HASP and SHERP expression from replacement constructs in the sand fly could include a role for vector-derived regulatory factors. To further investigate this hypothesis, selected *L*. *(L*.*) major* lines were incubated either with (+) or without (-) homogenized midguts of uninfected blood-fed sand flies harvested at day 6 and 12 PBM. Parasite growth was monitored every 24 h and lysates harvested at day 6 p.i. were immunoblotted and analysed using ImageJ. This analysis showed that the addition of midgut homogenate affected parasite growth in culture (Fig 9A). All tested lines grew more slowly with day 6 PBM midgut homogenate as compared to negative controls, failing to reach stationary phase by day 6 p.i. Conversely, parasite growth rates with day 12 PBM midgut homogenates were comparable to negative controls until day 3 p.i. when the negative controls reached stationary phase with subsequent decline in cell numbers. By contrast, the day 12 homogenate-supplemented parasite population continued to expand slowly until day 6 p.i. All tested lines showed a significant increase in growth when day 12 homogenates were compared to day 6 homogenates (FVI: P = 0.027; cDNA16 dKO: P = 0.016; cDNA16 sKI: P = 0.013; HASPA2 sKI: P = 0.015). FVI and cDNA16 sKI grown with day 6 PBM homogenate also showed a reduction of detectable HASPA and HASPB compared to the negative control (FVI: 2.7-fold and 1.1-fold; cDNA16 sKI: 2.2-fold and 3.8-fold, respectively; Fig 9B and 9C). Cultures grown with day 12 midgut homogenate showed more limited reduction in HASPA and HASPB levels compared to the negative control (FVI: 1.5-fold and 1.1-fold; cDNA16 sKI 1.4-fold and 1.2-fold, respectively), although these differences could be a consequence of the observed differences in parasite growth and potential slowing of metacyclogenesis. HASPA2 sKI and HASPB sKI were also tested in this way (Fig 9D). Compared to FVI and cDNA16 sKI, HASPA2 sKI showed a similar response in cultures spiked with day 6 and 12 PBM midgut homogenates, while HASPB sKI showed increased HASPB expression in response to both midgut homogenates (Day 6 PBM: 1.45-fold; Day 12 PBM 1.2-fold).

### The cDNA16 locus is required for parasite transmission

Since completion of metacyclogenesis, PSG plug formation and SV degradation have been hypothesised to be essential for successful parasite transmission, we wanted to experimentally confirm the failure of our metacyclogenesis-impaired mutant lines to be transmitted *in vivo*. Due to the complexity of these experiments, we were limited to testing only a small subset of mutants: cDNA16 dKO and HASPB sKI (both unable to complete metacyclogenesis, produce PSG or colonize the SV in *P*. *(P*.*) duboscqi*) as representative lines hypothesised to be non-transmissible; and cDNA16 sKI and FVI (competent for metacyclogenesis, PSG production and SV colonisation) as parasite lines predicted to be successfully transmitted to a suitable host.

**Fig 10:**
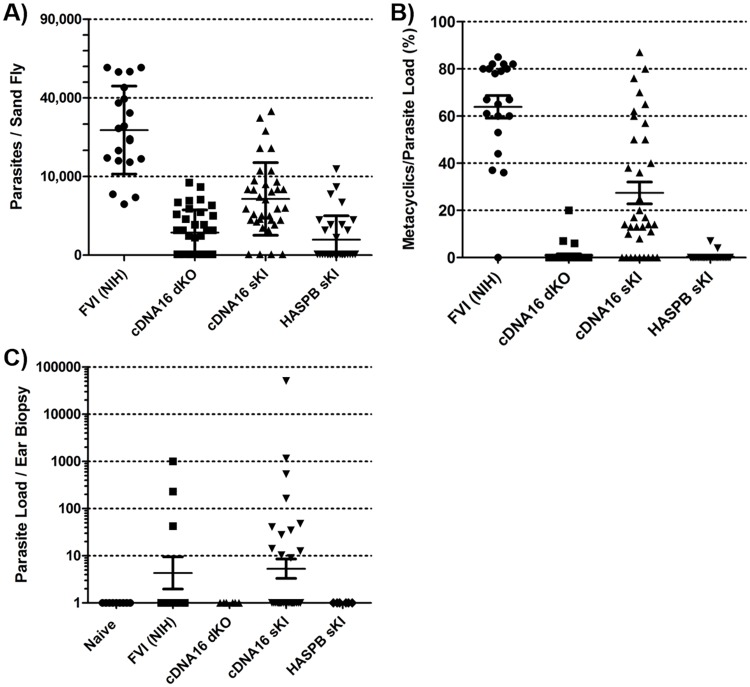
Parasite transmission from infected sand flies to naïve mice. A) Parasite loads were determined by releasing individual midgut contents into fixing solution on a haemocytometer under a light microscope. Parasite counts of the mutant lines were low in P. (P.) duboscqi midguts compared to FVI (NIH). cDNA16 sKI produced the most efficient mutant infections, although weaker than previously observed in Prague. cDNA16 dKO only produced comparatively low infections and HASPB sKI equally well as in previous observations in Prague. B) Morphometric evaluation of metacyclics present in the sand fly midgut. Metacyclics were identified on the haemocytometer by visual analysis during counting. Percentages of metacyclics within the whole count are represented as indicators of metacyclogenesis completion. Only FVI (NIH) and cDNA16 sKI produced metacyclics efficiently, as expected, although less efficiently than previously observed. C) cDNA16 sKI were successfully transmitted from day 14 PBM infected sand flies to naïve mouse ears in 37.5% of analysed biopsies. cDNA16 dKO and HASPB sKI were tested only in one of three repeats, but neither line showed successful transmission, like the uninfected sand fly control (Naïve). FVI (NIH) were detected in 27.3% of ear biopsies post infected sand fly exposure.

These experiments were conducted at the National Institute of Health (NIH), USA, using *P*. *(P*.*) duboscqi*, the same vector species used at the Charles University in Prague, CZ. Due to technical issues, the original FVI line was replaced with the FVI line available at the NIH (FVI (NIH)); both FVI lines are derived from the same original parent. Infection quality and metacyclogenesis progression in sand flies were monitored by parasite counting and morphometry, using light microscopy over a 14 day course PBM. FVI (NIH) presented similar infection loads (Fig 10A) as previously observed by qPCR for *P*. *(P*.*) duboscqi* in Prague (Fig 3B). While showing on average lower infection loads compared to FVI, HASPB sKI also showed comparable results between previous qPCR data and dissection (Figs 3B and 10A). cDNA16 dKO and cDNA16 sKI showed weaker infections compared to previous qPCR results (Figs 3B and 10A). The frequency (%) of metacyclics per midgut parasite load was also assessed, showing that only FVI (NIH) and cDNA16 sKI produced metacyclics efficiently in the vector (Fig 10B), as previously observed in the *P*. *(P*.*) duboscqi* colony from Prague (Fig 6), although FVI (NIH) was more efficient than cDNA16 sKI.

For the transmission experiments, sand flies infected with FVI (NIH), cDNA16 dKO, cDNA16 sKI or HASPB sKI were exposed on day 14 PBM to both ears of anaesthetised naïve mice. Genomic DNA was collected from the exposed ears and subjected to qPCR for parasite detection. As expected, the results showed successful parasite transmission for cDNA16 sKI and FVI (NIH) lines (37.5% and 27.3%, respectively, of analysed ear biopsies positive for parasite DNA), while cDNA16 dKO and HASPB sKI showed no evidence of transmission (Fig 10C). Interestingly, cDNA16 sKI was more efficiently transmitted than the FVI (NIH) control, despite the higher parasite loads and metacyclic content detected on average in FVI (NIH). This may relate to the higher virulence of cDNA16 sKI infections in mice (Fig 2A and 2B). However, for successful transmission, the arbitrary nature of sand fly feeding under these experimental conditions must also be considered. Since feeding success does not correlate with transmission success, this variable is difficult to quantify [40]. Either way, previous use of the FVI (NIH) line in this setting suggested that transmission success is commonly higher and comparable to cDNA16 sKI.

## Discussion

This study builds on our previous findings that the cDNA16 locus is essential for completion of metacyclogenesis in the sand fly midgut and that episomal expression of a HASPB gene copy can partially rescue the parental phenotype [30]. Here, we aimed to determine whether metacyclogenesis requires one gene from the cDNA16 locus, a subset of the HASP and/or SHERP genes, or the whole cDNA16 locus under parental gene regulation signals. Our in depth analysis has demonstrated a more complex picture.

The mRNA data reported here suggest that correctly regulated HASPB expression is the important event in metacyclogenesis in *L*. *(L*.*) major*, with cDNA16 sKI showing a similar expression pattern as FVI *in vivo* (Fig 8). This interpretation is further supported by the observed differences in HASPA and SHERP expression between the FVI and mutant lines, including cDNA16 sKI, suggesting that these genes are less likely to be required for completion of metacyclogenesis. The high expression of HASPB protein in *L*. *(L*.*) major* metacyclics [23] and the observation by Sádlová *et al*. (2010) that unregulated episomal expression of HASPB promoted metacyclogenesis completion further support this hypothesis [30]. Our data cannot directly prove that HASPB is the key player in metacyclogenesis *in vivo*, however, as the HASP and SHERP genes re-integrated alone into the cDNA16 locus are not expressed in the sand fly midgut. This is in contrast to the *in vitro* data collected during characterization of the same mutant lines, which show convincingly that the HASP and SHERP constructs are expressed and stage-regulated as in wild type parasites. Interestingly, replacement of the whole cDNA16 locus into its former location does recover the parental line phenotype *in vivo*, including detectable HASPB and SHERP product expression (Fig 7), while the step-by-step replacement of HASP and SHERP genes does not (see HA1/2+S2/HB sKI), suggesting that either the order and genomic context of the HASP and SHERP genes are vital to their correct regulation *in vivo* but not *in vitro*, or that trans-factors present *in vivo* play a role in parasite gene regulation in the sand fly.

A confounding factor here is the observed weak correlation between mRNA and protein abundance in *Leishmania species* [41]. In trypanosomes, mRNAs can be stored in cytoplasmic RNA granules until translation, potentially accounting for this discrepancy [42]. Recently, formation of mRNA granules has also been described for *Leishmania* under stress conditions, such as starvation [43], previously demonstrated to be a trigger for metacyclogenesis *in vitro* [6,7]. In the case of stage-regulated genes in *Leishmania*, as in other protozoan species [44], mRNA up-regulation can occur at a developmental stage prior to protein expression [41]. Although not definitively shown in *Leishmania*, it is feasible that these mRNAs are stored in RNA granules prior to entry into the translational machinery. Such sequestration could accelerate adaptational responses in dynamically changing environments, such as in the sand fly midgut, thereby promoting parasite survival. It is not known whether HASP and SHERP mRNAs are stored in RNA granules nor what signals might operate *in vivo* to initiate their subsequent translation. However, one testable hypothesis might propose that HASP and SHERP mRNAs are indeed sequestered cyoplasmically and “bypassed” *in vitro* due to a lack of midgut-specific signals in culture, thereby explaining the observed differences in HASP and SHERP mRNA and product expression *in vitro* and *in vivo*.

The above observations suggest that HASP and SHERP function could be related to cellular mechanisms required for survival and development under sand fly midgut conditions. In this dynamic environment, alterations in pH, temperature, amino acid and digestive enzyme content occur during parasite differentiation, changes that are only partially reproduced under culture conditions. The importance of *in vivo* passaging to restore virulence in *L*. *(L*.*) major* and other *Leishmania spp*. has been previously demonstrated [40,45,46]. Prolonged *in vitro* culture frequently causes parasite avirulence [47], which can be partially restored by repeated passaging through a susceptible mammalian host or sand fly vector [45]. Similar effects have been reported in other parasitic systems: culture-adapted *Trypanosoma brucei* bloodstream parasites, for example, have a 1,000x lower antigen switching rate for their variable surface glycoproteins (VSG) than in natural isolates [48,49]. Interestingly, the addition of crude uninfected blood-fed midgut homogenates to cultured *Leishmania* promastigotes in this current study indicated potential regulatory and temporal influences on parasite growth and HASPA and HASPB expression in the period following the blood meal time (Fig 9). Considering that the midgut is subject to dynamic changes over the course of *Leishmania* infection, reflecting the transition from blood meal to sugar digestion, our data suggest that *Leishmania* could utilise changes in the midgut content to drive its gene regulation and subsequent proliferation and differentiation. Well-established examples of this type of parasite/host interaction can be found in other kinetoplastid species such as *T*. *brucei*, during differentiation into stumpy forms [50,51] and trypomastigotes [52–54] and during gametocytogenesis in *Plasmodium spp*. [55].

It is also possible that differences in mutant gene expression are chromosomal context dependent. The observation that the *Lmj*cDNA16 sKI line is the only one of the range of mutants generated to express HASPB and SHERP *in vivo* (Fig 7) and rescue metacyclogenesis (Figs 4 and 6), as described above, suggests that the order and context in which the HASP and SHERP genes occur in the cDNA16 locus are relevant for correct *in vivo* regulation. Accurate polycistronic RNA processing for the production of mature mRNAs in *Leishmania* requires the correct positioning of downstream gene 5’-splice acceptor sites relative to the upstream gene polyadenylation sites [56]. Clearly our constructs, with their flanking sequences and DHFR-flanked antibiotic-resistance genes, are expressed appropriately *in vitro* but not *in vivo*, suggesting that other factors are important in regulating expression from our constructs in the sand fly midgut. While further investigation into HASP and SHERP function is highly desirable, the lack of HASP and SHERP mutant phenotypes *in vitro* hampers further rapid investigation due to the complexity of parasite manipulation in the sand fly midgut and the limiting amounts of biological material recoverable from infected sand flies.

Sádlová *et al*. [30] showed that cDNA16 dKO parasites grown *in vitro* secrete fPPG, constituent of the PSG, into the culture medium. By contrast here, only FVI and cDNA16 sKI secrete detectable amounts of fPPG in the sand fly midgut (Fig 5C) leading to formation of PSG that is detectable by light microscopy (Fig 5B). While further analysis using a panel of relevant antibody probes would be desirable, our current data suggest that PSG is not produced in the cDNA16 mutant lines *in vivo*. It is unclear why fPPG secretion and PSG formation should be impaired *in vivo* in the sand fly midgut but not *in vitro*, and how the HASPs and SHERP contribute to this process. The PPG synthetic pathway, while not completely described in *Leishmania*, involves secretion via the ER and Golgi in other eukaryotic cells [57], two cellular compartments that the HASPs and SHERP do not appear to enter. Rather, N-myristoylated HASPs are palmitoylated on the cytosolic face of the Golgi, prior to transport to the plasma membrane [20], while SHERP associates intracellularly as a peripheral membrane protein [27], interacting *in vitro* with a sub-unit of a vacuolar type H(+)-ATPase that functions in acidification [28]. There is no evidence to suggest that SHERP affects secretion via the ER/Golgi route, but it cannot be excluded that this small protein influences fPPG synthesis and/or secretion indirectly by its interactions with intracellular membrane constituents [27]. Alternatively, the lack of fPPG secretion could be a consequence of incomplete metacyclogenesis in the HASP and SHERP mutants preventing maturation of fPPG-secreting leptomonads. This seems unlikely, given that leptomonad forms were observed by morphometry in comparable numbers in HA1/2+S2/HB sKI, FVI and cDNA16 sKI, while PSG formation in the TMG was only observed in FVI and cDNA16 sKI infections. Since there are no molecular markers for mature leptomonads, it cannot be excluded that the parasite forms observed by morphometry were functionally immature, however. Further investigation is required to determine the relationship between HASP and SHERP deletion and the lack of PSG formation.

PSG has been shown to play a role in TMG colonization in the sand fly [13,58], which could explain why several mutant lines lacking fPPG failed to establish mature infections and to accumulate in the TMG, in particular, in *P*. *(P*.*) papatasi*. The PSG plug forces sand flies to regurgitate prior to blood meal intake, supporting parasite transmission into the host skin [38]. Once in the skin, glycans donated by the fPPGs promote the recruitment of neutrophils and macrophages [38], prior to macrophage invasion [59,60]. Further, PSG induces the alternative activation of macrophages, promoting arginase-1 activation and antagonising nitric oxide synthase 2 (NOS2), thereby facilitating parasite survival [60]. Therefore, a lack of PSG may impair sand fly transmission and reduce the likelihood of parasite survival in the mammalian host. While we were able to show impaired transmission in our PSG-deficient mutant lines *in vivo*, the same lines cultured *in vitro* were all able to establish persistent infection in BALB/c mice following sub-cutaneous high dose needle infection of 10^7^ late-stage parasites. Interestingly, several PSG-deficient lines, in particular cDNA16 dKO, HASPA1 sKI and HASPA1/2 sKI, were more virulent in BALB/c mice than FVI, while other lines were significantly less virulent (e.g. HASPA2 sKI). However, it must be noted that *in vitro*, these parasite lines were able to complete metacyclogenesis and to secrete fPPG [30]. *In vivo*-derived parasites from these PSG-deficient lines show a lack of metacyclic generation, suggesting that parasites would not be infective, even if they were transmitted from the sand fly to a mammalian host. Since our data showed that our mutant lines are not transmissible, with the exception of cDNA16 sKI, this question could not be addressed in a natural transmission scenario. Even so, our results confirmed experimentally that completion of metacyclogenesis, PSG secretion and SV colonization, which are all hallmarks of mature parasite infections in sand flies, are essential for successful parasite transmission, as is the presence of the cDNA 16 gene locus.

On artificial challenge in BALB/c mice with our mutant lines, significant differences were observed in pathology between mutant lines. As previously observed by McKean *et al*. (2001), the full cDNA16 locus deletion mutant (cDNA16 dKO) caused faster footpad swelling than the parental line (FVI; Fig 2C). Interestingly, the phenotype of the full cDNA16 locus replacement line (cDNA16 sKI) was intermediate, suggesting gene dose dependency of the observed phenotype. Since HASPA1 sKI has a similar phenotype to cDNA16 sKI, while HA2+S2/HB sKI has a similar phenotype to FVI, this suggests that the cDNA16 sKI intermediate phenotype is HASPA1 gene dependent. Although not directly demonstrated, these data suggest that the deletion of the cDNA16 locus products increases inflammatory responses (either directly or by pleiotropic effects), a phenotype moderated by re-introduction of one copy of the locus containing half the wild type copy number of HASP and SHERP genes. This hypothesis is supported by the observation in our previous study [31] that an episomal cDNA16 replacement mutant, overexpressing HASP and SHERP, was avirulent. While HASPA1 sKI showed a similar phenotype as cDNA16 sKI, the HASPA2 sKI mutant line showed a significantly delayed onset of lesion formation, despite equivalent inoculum and *in vitro* capacity for metacyclic formation (Fig 2A and 2B). This observation was of interest because HASPA2 protein expression is promastigote-specific, while HASPA1 protein expression is amastigote-specific (Figs 1D and 2E), correlating with the mRNA expression data for these genes [17]. Introducing a HASPA1 copy with a HASPA2 copy (HASPA1/2 sKI) caused a similar phenotype as HASPA1 sKI (Fig 2C). Interestingly, the parasite burden per footpad at 2 mm swelling was similar for all tested lines; only the time point when these were reached varied between the lines. These observations could indicate functional differences in the HASPA proteins during the parasite life cycle, potentially affecting initial metacyclic parasite survival post inoculation or amastigote proliferation rather than disease progression per se; a surprising conclusion given their identical protein sequences.

Based on the results presented here, we confirm a role for both the HASP and SHERP proteins in *Leishmania* metacyclogenesis in the sand fly, leading to parasite transmission, and propose a subsequent role for the HASP proteins in establishing infection in the mammalian host. We also show for the first time that sand fly midgut extracts collected post-blood meal affect parasite behaviour *in vitro* over time as the midgut luminal content changes. While biochemical fractionation and in depth analysis of the midgut lysates are now required to advance these studies, they may offer a novel approach to simulate *in vivo* phenotypes *in vitro* while also affirming the importance of vector-parasite interactions *in vivo*.

## Materials and Methods

### Parasites and mutant generation

*L*. *(L*.*) major* Friedlin V1 (MHOM/IL/81/Friedlin/VI; FVI) was used as a parental line/wild type in this study, as for all previous work on this locus. The previously described *L*. *(L*.*) major* 4.8 cDNA16 double deletion (cDNA16 dKO; *ΔcDNA16*::*HYG/ΔcDNA16*::*PAC*; [31]) and cDNA16 single replacement lines (cDNA16 sKI; *ΔcDNA16*::*HYG/ΔcDNA16*::*PAC/ΔPAC*::*cDNA16+ NEO* [30]) were used as controls. New *L*. *(L*.*) major* HASP and SHERP replacement lines were generated by homologous recombination with newly synthesized linear DNA constructs into the former cDNA16 locus within the null or other mutant backgrounds, using the nucleofection kit (Amaxa) according to the suppliers’ guidelines. The DNA constructs contained one or two HASP and/or SHERP gene(s) with their native 5’ and 3’ UTRs and a selectable antibiotic resistance marker gene (either NEO or BSD). Correct genomic integration of DNA constructs was verified by Southern blot and quantitative PCR (qPCR) followed by parasite passage through BALB/c mice (described below). Promastigotes of all *L*. *(L*.*) major* lines were routinely cultured in Medium 199 (M199) (supplemented with 20% v/v heat-inactivated foetal calf serum, 1% v/v penicillin-streptomycin) as described [31]. For parasite *in vitro* differentiation, parasites grown until late log-phase in M199 were harvested, washed and suspended in 5% sucrose/PBS (described below).

Metacyclic purification by peanut agglutination (PNA; Sigma) was originally described elsewhere [61]. We used 50 μg/ml PNA for 15 min. at room temperature with regular agitation of parasite suspensions to separate agglutinated promastigote forms from metacyclics by slow centrifugation (175x*g*). Metacyclic ratios in day 7 cultures were established by pre- and post-agglutination counts on a haemocytometer.

### DNA analysis

The protocol for Southern analysis has been described elsewhere [30]. Genomic DNA (gDNA) was extracted using DNeasy Blood & Tissue columns (Qiagen), *Sac*I-*digested*, separated by 0.8% agarose gel electrophoresis and blotted onto positively charged nitrocellulose membranes. Digoxigenin (DIG) labelled probes were used for detection with the DIG-development system (Roche).

### Immunoblotting

Whole parasite lysates in Laemmli buffer (20 ml 0.5 M Tris-HCl [pH 6.8], 3.08 mg DTT, 40 ml SDS [10%], 50 mg Bromophenol Blue, 20 ml Glycerol [100%] and sterile Milli-Q water (MQH_2_O) to 100 ml) were separated by SDS-PAGE as described [31] and blots analysed using affinity-purified polyclonal rabbit antibodies against SHERP [27], HASPB (336; [31]) or non-affinity-purified polyclonal HASP antibodies for HASPA-detection. A secondary anti-rabbit HRP antibody (Sigma) was used with ECL Prime (Amersham) for detection. A polyclonal antibody against *L*. *(L*.*) major N*-myristoyltransferase (NMT; [36]) was used as a loading control. In case of the growth condition assay, band intensity on immunoblots were analysed using ImageJ. Values were normalized first for each gene individually (HASPA2, HASPB and NMT, respectively) against negative control to compensate variations within the image. Then normalized HASPA and HASPB values were normalized against the corresponding NMT loading control to adjust for loading variations prior to calculating the fold-difference between (+) and (-) conditions.

PSG was extracted from 10 infected midguts pooled into 50 μl PBS, as described [60]. Whole lysates of debris pellets and 6x spun PSG containing supernatants were blotted on to activated nitrocellulose membranes using an adapted protocol [60]. Dot blots were probed for PSG with the LT15 antibody against phosphoglycan disaccharide repeats [PO4-6Galb1-4Mana1-]x [38] and treated as for the immunoblots using an anti-mouse HRP secondary antibody (Sigma).

### Osmotaxis assay

The osmotaxis assay was adapted from Leslie *et al*. [62] and Oliveira *et al*. [63]. Briefly, plain glass capillary tubes (75 mm length, 0.8 inner/1 mm outer diameter) were filled with wash and incubation (WIS) buffer (30 mM β-glycerophosphate disodium salt, 87 mM NaCl, 27 mM KCl, 2 mM CaCl2, 2 mM MgCl2, 0.004% enriched Bovine Serum Albumin [pH 7.1]) containing 1% agarose ± 100 mM of sucrose, leaving exactly 1 cm void (~5 μl). Once set, the void was filled with WIS buffer. The glass capillaries were equilibrated in WIS buffer for ~30 min. at room temperature on a rocking table. Parasites were grown to late log-phase/early stationary-phase in M199, harvested, washed twice in WIS buffer and suspended to a final concentration of ~2.5x10^7^ cells/ml in WIS buffer. The equilibrated glass capillary tubes were dipped into the parasite suspension at a slight angle (6 capillary tubes with 100 mM sucrose and 6 without sucrose were used per strain) and incubated at 26°C for 1 h. The WIS buffer in the capillary void was removed, mixed with 1% formaldehyde in saline solution and applied to a haemocytometer for parasite counting.

### Sand fly infections

At the Charles University, Prague, sand flies were maintained at 26°C and high humidity (75%) on 50% sucrose solution and a 14 h light/10 h dark photoperiod as described [64]. Sand fly infections and analysis were carried out as described by Sádlová *et al*. [30]. For infections, colony-bred *Phlebotomus (Phlebotomus) papatasi* and *P*. *(P*.*) duboscqi* (Turkey and Senegal strains, respectively; reared at the Department of Parasitology, Charles University, Prague) were fed on infected (10^6^ parasites/ml) heat-inactivated rabbit blood through a chick-skin membrane for up to 2 h in the dark. Unfed sand flies were separated from engorged females, which were sampled at day 2, 5, 9 and 12 post blood meal (PBM) or at day 6 and 12 PBM only. Light microscopic analysis of dissected midguts was used to establish parasite localization (endoperitrophic space, AMG to cardia, attached to the stomodeal valve either weakly or strongly) and parasite load per midgut for all dissection points; scoring adapted from Myšková *et al*. [65] as light (<100 parasites/gut), moderate (100–1000 parasites/gut), heavy (>1000 parasites/gut) and very heavy (>>1000 parasites/gut). Dissected midguts were scored by two independent researchers. In addition, 30 infected female sand flies from day 12 PBM were individually frozen at -20°C in buffer for DNA extraction and parasite loads were scored by qPCR as described [30]. Sand fly infection experiments were repeated at least three times per tested line.

At the National Institute of Health (NIH), Rockville, USA, sand flies were maintained in similar conditions. For infection, colony-bred 2- to 4-day-old *P*. *(P*.*) duboscqi* females (Mali strain; reared at the Laboratory of Malaria and Vector Research, NIAID) were infected by artificial feeding through a chick skin membrane on defibrinated rabbit blood (Spring Valley Laboratories, Sykesville, MD) containing 350 units/ml penicillin, 350 μg/ml streptomycin, and 3–4 x 10^6^ procyclics or amastigotes/ml (P1-P5) from four *L*. *major* lines (FVI, cDNA16 dKO, cDNA16 sKI and HASPB sKI) initially isolated from BALB/c footpad lesions. After 3 h of feeding in the dark, fully blood-fed sand flies were separated. For the initial experiment, the total number of parasites and percent metacyclics per midgut were established at different days after infection (D2, D7, D9 and D14; data on for D14 shown in Fig 10) for each group. Thereafter, in two independent repeats, the total number of parasites and the percentage of metacyclics were determined on the day of transmission. After dissection, each midgut was placed in 50 μl of PBS in a microcentrifuge tube, macerated with a plastic pestle (Kimble Chase) and parasites counted using a haemocytometer; metacyclic forms were distinguished by morphology and movement [66].

### Quantitative PCR

Quantitative PCR was performed on genomic DNA samples of selected new mutant clones to establish integrated copy number using Fast SYBR Master Mix (Applied Biolscience) in the OneStep qPCR system (Life technologies) with the OneStep software v.2.2.2 according to the supplier’s guidelines. Target genes were detected with gene-specific primers and results were normalized against the Na/H antiporter-like protein gene (see S6 Table). The protocol for detection of parasite load is described elsewhere [30,65]. Primers specific for kinetoplast minicircle DNA were used for parasite detection as described by Mary *et al*. [67] (S6 Table).

### In vitro simulation of in vivo parasite growth conditions

Parasites were grown for 3 days in 10 ml complete M199 at 26°C, pelleted and washed twice in sterile PBS at room temperature. Parasites resuspended in 10 ml 5% sucrose/PBS were incubated for an additional 4 days at 26°C. 10^7^ parasites were collected every 24 h and lysed in Laemmli buffer for Western analysis.

Sand flies were fed on heat inactivated uninfected rabbit blood through a chick-skin membrane for up to 2 h in the dark. 50 blood fed midguts were dissected at day 6 and 12 PBM into 200 μL M199 + Amikin (250 μg/ml) + penicillin (60 μg/ml) + fluorocytosin (1.5 mg/ml). The midguts were homogenised and filter through a 0.22 μm filter spinning column (Ultrafree—MC, GV dutapore). The midgut extract was added to 4 ml of M199 and 1 ml was aliquoted into culture tubes. M199 was chosen as the medium of choice given its high serum content, which reduced the impact of additional protein introduced via the midgut homogenate. FVI, *Lmj*cDNA16 dKO, *Lmj*cDNA16 sKI and *Lmj*HASPB sKI were inoculated into the 1 ml medium, respectively, to a final concentration of 10^5^ parasites/ml and left to grow for 6 days. Parasite density was established by parasites counting on a haemocytometer. At day 6 p.i., parasites were pelleted and washed twice in PBS before lysis in 50 μl 1x Laemmli buffer at 95°C for 10 min.

### Parasite morphometry and immunofluorescence analysis

As described previously [30], midguts were divided into AMG and TMG, smeared on to glass slides, fixed with 100% methanol and stained with Giemsa. 160 randomly chosen parasites were imaged (using a 100x oil-immersion objective, Olympus BX51 fluorescent microscope, Olympus DP70 camera) per midgut section per day per strain on at least three randomly chosen gut smear slides. For cultured parasites, cell pellets were washed twice in saline solution (0.9% NaCl), applied to polylysine slides for 15 min. and the excess suspension tipped off. Glass slides were air dried, fixed with 100% methanol, rinsed with water, stained with Giemsa and analysed by microscopy. 100 randomly chosen parasites per slide were imaged (using a 63x oil-immersion objective, Zeiss Axioplan microscope, Optronics 60800 camera system).

For immunofluorescence analysis, cultured parasites were harvested, washed, fixed in 4% formaldehyde and applied to polylysine glass slides as described [21,68]. Parasites derived from sand flies were analysed in 100% methanol fixed gut smears as above. Cells were permeabilized with 0.2% Triton-X 100/PBS and blocked with Image-iT FX signal enhancer (Invitrogen) prior to analysis of HASPB or SHERP expression using polyclonal anti-HASPB (336; [32]) and anti-SHERP [27] antibodies, respectively, followed by Alexa Fluor 488 Dye (Invitrogen) secondary antibody. Samples were mounted either in Vectashield or Mowviol with 4', 6-diamidino-2-phenylindole (DAPI; Vector) and imaged with Zeiss LSM 510 or 710 META confocal microscopes.

Flagellum and cell body length and width were measured with Image J [69]. Parasites were classified into four groups adapted from Walters (1993) and Ciháková and Volf (1997): (i) procyclics < 14 μm body length ≥ 4 μm, flagellum shorter than cell body; (ii) nectomonads: body length ≥ 14 μm; (iii) leptomonads: body length < 14 μm and flagella length < 2 times body length and (iv) metacyclics: body length < 14 μm and flagella length ≥ 2 times body length. Paramastigotes and haptomonads were not considered. *In vivo*, haptomonad presence or absence was determined by the state of SV colonization.

### Parasite infections

Experiments routinely used 6–8 weeks old BALB/c mice (Harlan Laboratories, UK). For footpad infections, 3x 10^7^ late-stage stationary parasites were injected in 30 μl PBS (adapted from Depledge *et al*. [70]). For routine parasite passage to re-establish virulence, amastigotes were harvested from draining lymph nodes 8–10 weeks post footpad infection and inoculated into M199 for incubation at 26°C.

To establish parasite virulence, footpads of groups of 5 female BALB/c mice were infected, ears were marked and mice placed randomly in cages. Mice were chosen blindly for footpad measurement once a week until footpad lesion reached 2 mm, at which point they were sacrificed. Parasite burdens per footpad were established after mouse-scarification by a limiting dilution assay (LDA) of infected footpads, adapted from Titus *et al*. [71] and Lima *et al*. [72] and calculated by the online *Leishmania* LDA analysis tool available on the Imperial College London homepage: (http://wwwf.imperial.ac.uk/theoreticalimmunology/llda/) [73].

Amastigotes were isolated using a protocol adapted from Paape and Aebischer (2011)[74]. BALB/c mice were infected at the base of the tail on both sides by needle inoculation of 3x10^7^ late stationary parasites. After lesions developed (8–10 weeks p.i.), mice were sacrificed and lesion material excised with a scalpel, weighed, forced through a 70 μm cell strainer into homogenization buffer (20 mM HEPES-KOH, pH 7.3, 0.25 M sucrose supplemented with cOmplete Mini proteinase inhibitor cocktail [Roche]) and washed once in homogenization buffer. Amastigotes were released by forcing the cell suspension through a 25-gauge needle. Nuclei were removed by centrifugation at 100x*g* for 2 min. The supernatants were loaded onto a discontinuous sucrose gradient: 20, 40, and 60% (w/w) sucrose in HEPES saline (30 mM HEPES-KOH, pH 7.3, 0.1 M NaCl, 0.5 mM CaCl_2_, 0.5 mM MgCl_2_) [75], centrifuged for 25 min. at 700x*g*. Amastigotes were isolated from the 40/60% sucrose interface, diluted in PBS and washed once in PBS.

### Parasite transmission in vivo

Experiments at the NIH were performed using 6- to 8-week-old BALB/c mice (Charles River Laboratories Inc.), maintained under pathogen-free conditions. To assess the capacity of mutants to transmit to BALB/c mice, 10–20 (as available) infected *P*. *(P*.*) duboscqi* females from each group (FVI, cDNA16 dKO, cDNA16 sKI and HASPB sKI) on day 14 PDM were exposed to each mouse ear in the first experiment. Thereafter, validation of transmission was carried out for the groups where mature infections containing metacyclics were observed (FVI and cDNA16 sKI). Mice were anesthetized intraperitoneally with a mixture of ketamine (100 mg/kg) and xylazine (10 mg/kg). Infected sand flies were placed in vials with a meshed surface and applied to the ears using custom-made clamps. The flies were allowed to feed for two hours in the dark. Mouse ears were removed two hours after exposure to infected flies and frozen at -70°C until processed. Total genomic DNA was extracted using the DNeasy tissue kit following the manufacturer’s protocol (Qiagen). A total of 50 ng of sample DNA was amplified in triplicate by real time PCR (Biorad c1000 thermal cycler and cfx96 real time system) using primers JW11 and JW12 [76] together with a Ld3C6 fluorescent probe [77] targeting kinetoplast minicircle DNA. Parasite numbers were determined by the real time system software based on a standard curve of serially diluted *L*. *major* (WT Friedlin V1) DNA. The cut-off was based on values obtained for naïve DNA controls.

### Ethics statement

Animal experiments in York were approved by the University of York Animal Procedures and Ethics Committee and performed under UK Home Office license (‘Immunity and Immunopathology of Leishmaniasis’ Ref # PPL 60/4377). All animal experimental procedures at the NIH were reviewed and approved by the National Institute of Allergy and Infectious Diseases (NIAID) Animal Care and Use Committee under animal protocol LMVR4E. The NIAID DIR Animal Care and Use Program complies with the Guide for the Care and Use of Laboratory Animals and with the NIH Office of Animal Care and Use and Animal Research Advisory Committee guidelines.

### Statistical analysis

All statistical analysis was done with the SPSS software v.20-22 (IBM) or GraphPad Prism 5. P<0.05 was considered to be significant. Categorical data were analysed by *χ*^2^ test. Normally distributed continuous data were analysed by one-way ANOVA and *post-hoc* Tukey multiple comparison test. Non-normally distributed data were normalized by log_10_ or square root transformation, as appropriate, and submitted to parametric analysis where possible. Otherwise, non-normally distributed data were analysed by Kruskal-Wallis and *post-hoc* Dunn’s multiple comparison test. Growth assay and footpad lesion data were analysed by repeat measure ANOVA and *post-hoc* Tukey multiple comparison test or by Freidman test and *post-hoc* Dunn’s multiple comparison test.

## Supporting Information

S1 Fig HASP and/or SHERP replacement-construct plasmids. Schematic representation of all HASP and SHERP gene constructs used in this study within the pCR2.1-TOPO vector generated for homologous recombination into the *L*. *(L*.*) major* cDNA16 locus. Open reading frames (ORFs) are in orange, promoters in burgundy and all other elements in green. Arrows indicate reading orientation.
(TIF)

S2 Fig Targeted complementation of HASP and SHERP genes into the cDNA16 locus. Schematic representation of HASP and SHERP replacement constructs integrated into the former *L*. *(L*.*) major* cDNA16 locus in the different *L*. *(L*.*) major* mutant lines generated for this study. Refer to S1 Table, Figs 1A and S1 for more details on the replacement constructs.
(TIF)

S3 Fig PCR screen for replacement construct integration. PCR screening of transfected *L*. *(L*.*) major* mutant clones for construct integration allowed rapid large scale selection of clones for further analysis (A-E). A minimum of 20 clones, where available, were first screened in this way. Gel images are representative for clones analysed in this study only, each identified by (number of clones) in the construct name. The gene names following the (number of clones) in images (B-E) refer to the gene targeted in that lane. (F) This Table lists the expected sizes of PCR products generated with the named primer pairs and targeted constructs.
(TIF)

S4 Fig Southern blots of selected clones. Southern blotting was used to confirm correct integration of replacement constructs and to exclude clones containing episomal constructs. Blots were probed multiple times with different DIG-labelled probes for the construct genes, resistance markers and the 5’ flanking region required for integration. Varying numbers of clones per mutant line were screened for clone selection. The blots shown are representative of the clones presented in this study only; the blot in Fig 1B was derived from these data.
(TIF)

S5 Fig Integrated construct copy number analysis by qPCR in selected clones. This expanded analysis of Fig 1C shows multiple clones per mutant line analysed for replacement construct copy number after transfection. All results were normalized internally against the Na/H antiporter-like protein on chromosome 23 and against FVI.
(TIF)

S6 Fig Time-course immunoblot analysis of HASPA, HASPB and SHERP expression in selected mutant lines. This expanded analysis of Fig 1D shows a single representative clone for each mutant line (labelled A-R) tested in this study. Clone identifiers are shown in brackets. For mutant lines shown in Fig 1D the alternative clone is shown here.
(TIF)

S7 Fig Time-course immunoblot analysis of two HASPA1/2 sKI clones. Immunoblots of one further HASPA2 sKI (18) and two further HASPA1/2 sKI (16 & 18) clones are shown. The HASPA2 sKI clone shows a similar HASPA expression pattern in promastigotes as observed in FVI and the HASPA2 sKI clone in Fig 1D. The additional HASPA1/2 sKI clones are expressing high and unregulated levels of HASPA as the clone shown in Fig 1D. The HASPA1/2 construct contains the same DNA fragments as the HASPA1 and HASPA2 constructs, which did not show the same level of expression. Thus the strong HASPA expression in the HASPA1/2 sKI line is not a clonal artefact, but a conserved property of the mutant line.
(TIF)

S8 Fig Growth assay of selected clones. This expanded analysis of Fig 1E shows growth kinetics of selected clones of the different mutant lines. All clones were inoculated at 10^5^ parasites/ml into 10 ml culture medium 199 and grown at 26°C for 7 days. Parasite numbers were counted once a day on a haemocytometer. These growth assays show that genetic transfection had no adverse effect on the viability and proliferation capacity of *L*. *(L*.*) major in vitro*.
(TIF)

S9 Fig Infection Assay (Fig 2C and 2D split into respective clones, a, c and b, d). This figure shows the data presented in Fig 2C and 2D separated into the respective clones tested. a) and c) show the infection assay data by clone per line. Statistically significant differences (P<0.001) by repeat Friedman test measurements are observed for both HASPA2 sKI clones compared to all other lines/clones within the respective repeats. b) and d) show the LDA data by clone per line. b) represents the same clones as in a) and d) shows the same clones as in c). No statistically significant differences by Kruskal-Wallis test are observed for both HASPA2 sKI clones compared to all other lines/clones within the respective repeats. The clones shown in Figures a) and b) are HASPA1 sKI (3), HASPA2 sKI (18), HASPA1/2 sKI (8), HA2+S2/HB sKI (11). The clones shown in Figures c) and d) are HASPA1 sKI (8), HASPA2 sKI (16), HASPA1/2 sKI (18), HA2+S2/HB sKI (12).
(TIF)

S10 Fig Giemsa stained metacyclics isolated from culture. Three separate images are shown for each *L*. *(L*.*) major* line tested in three repeat agglutination assay experiments (refer to Fig 2B), showing Giemsa stained culture-derived metacyclics for subsequent morphometric verification. Size bar is equivalent to 10 μm.
(TIF)

S11 Fig Parasite infection intensities in sand fly midguts analysed by light microscopy. This expanded analysis of Fig 3A shows a single representative clone for all *L*. *(L*.*) major* mutant lines tested in sand flies.
(TIF)

S12 Fig Parasite infection intensities in sand fly midguts analysed by qPCR. This expanded analysis of Fig 3B shows a single representative clone for all *L*. *(L*.*) major* mutant lines tested in sand flies.
(TIF)

S13 Fig Parasite localization in the sand fly midgut over time. This expanded analysis of Fig 4 shows a single representative clone for all *L*. *(L*.*) major* mutant lines tested in sand flies. The *, “and ^ following the line names identify the separate sets of triplicate repeat experiments.
(TIF)

S14 Fig Morphology of sand fly midgut-derived promastigotes. This expanded analysis of Fig 6 shows a single representative clone for all *L*. *(L*.*) major* mutant lines tested in sand flies. The *, “and ^ following the line names identify the separate sets of triplicate repeat experiments.
(TIF)

S15 Fig Parasite growth in 5% sucrose. Immunoblot time course analyses of HASP and/or SHERP probed parasites (FVI, cDNA16 dKO, cDNA16 sKI, HASPB sKI and SHERP sKI) differentiated either in M199 or in 5% sucrose/PBS until day 7 p.i. The latter resembles more closely the nutrient depleted conditions during parasite differentiation in the sand fly midgut following blood meal defecation; parasites were transferred from M199 into 5% sucrose/PBS at day 3 p.i.
(TIF)

S1 Table Complete table of *Leishmania (Leishmania) major* mutant lines. This expanded version of Table 1 shows all *L*. *(L*.*) major* lines used in this study and included in the supplementary figures.
(TIF)

S2 Table Numbers of collected sand flies analysed in the *Leishmania* differentiation study. The table lists the total numbers of female sand flies dissected per line per day PBM; see Figs 3A and 4.
(TIF)

S3 Table Parasite localization in the midgut by percentage of infected midguts. Fig 4 results are presented as percentages of all sand flies analysed per mutant line. Refer to S1 Table for information on dissected sand fly numbers per respective *L*. *(L*.*) major* line tested.
(TIF)

S4 Table Parasite localization in *P. (P.) papatasi* midguts by percent of infected midguts. This expanded version of S3 Table shows all the *L*. *(L*.*) major* mutant lines infected into *P*. *papatasi*.
(TIF)

S5 Table Parasite localization in *P. (P.) duboscqi* midguts by percentage of infected midguts. This expanded version of S3 Table shows all the *L*. *(L*.*) major* mutant lines infected into *P*. *duboscqi*.
(TIF)

S6 Table qPCR primer table. (TIF)
