# Supplementary figures and images for: Leishmania HASP and SHERP Genes Are Required for In Vivo Differentiation, Parasite Transmission and Virulence Attenuation in the Host
Source: PLoS Pathog. 2017 Jan 17;13(1):e1006130. doi: 10.1371/journal.ppat.1006130 (PMC5271408; doi:10.1371/journal.ppat.1006130)

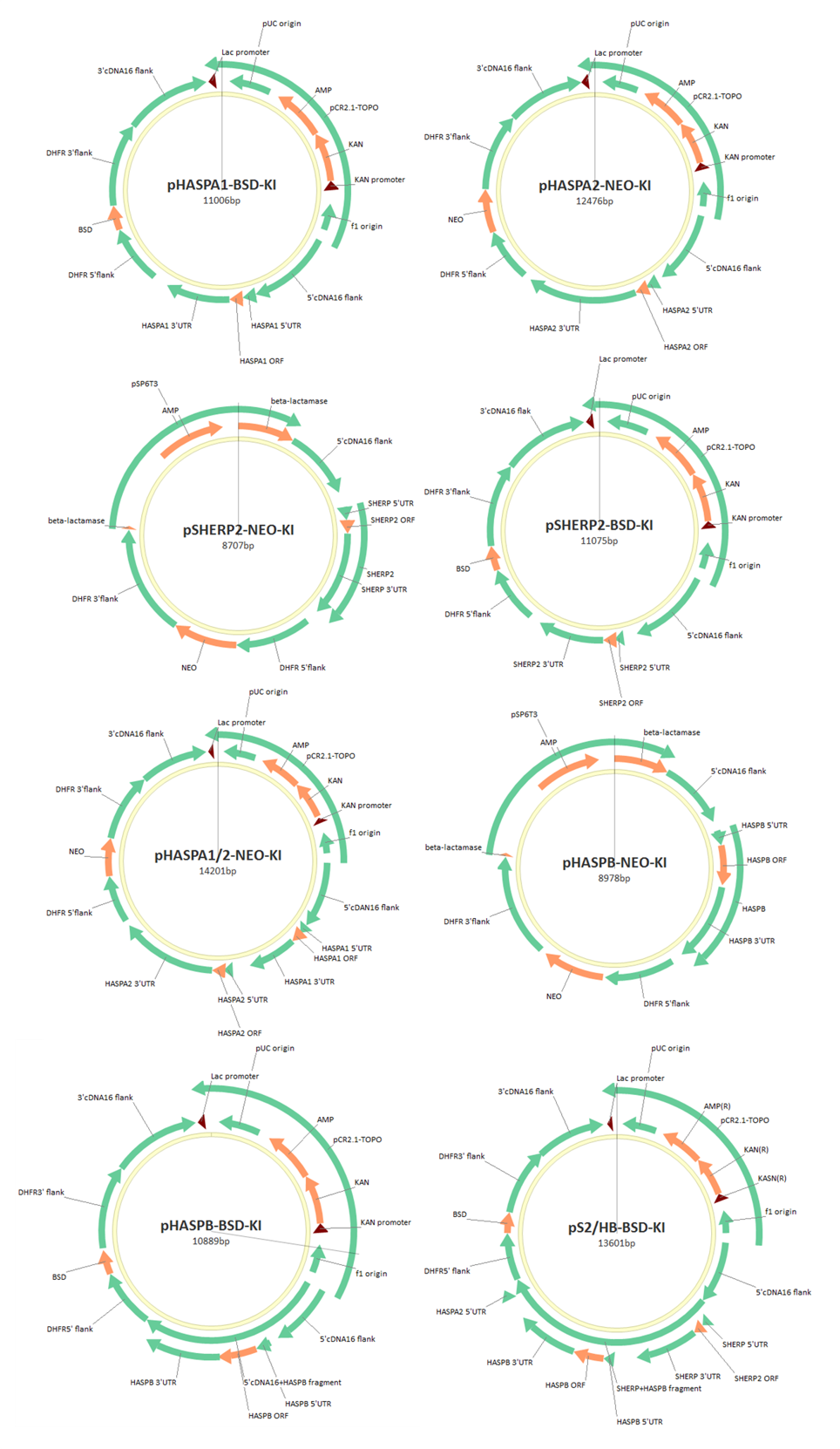

Supplement: S1 Fig — Schematic representation of all HASP and SHERP gene constructs used in this study within the pCR2.1-TOPO vector generated for homologous recombination into the L. (L.) major cDNA16 locus. Open reading frames (ORFs) are in orange, promoters in burgundy and all other elements in green. Arrows indicate reading orientation. (TIF) [file ppat.1006130.s001.tif]

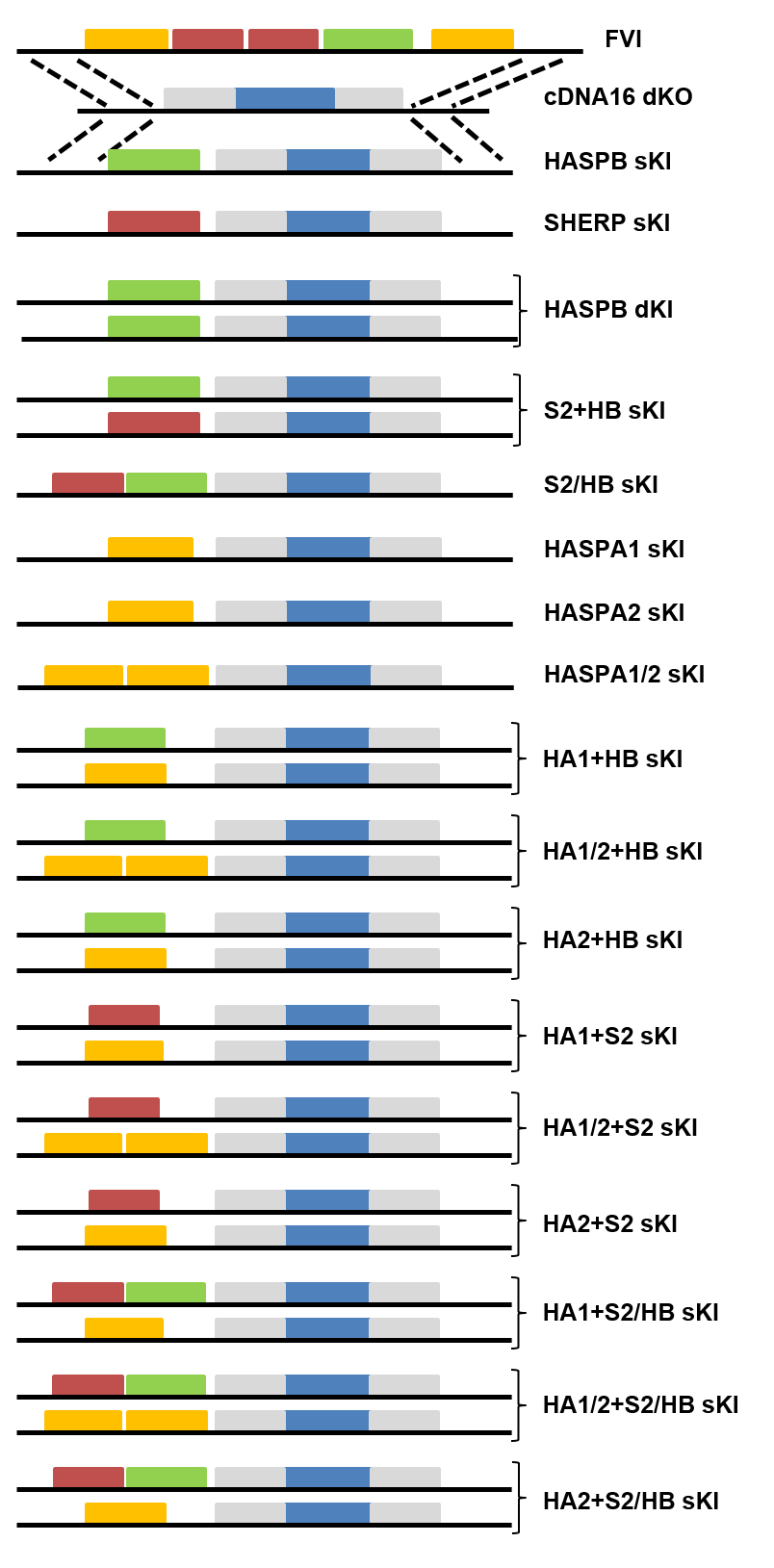

Supplement: S2 Fig — Schematic representation of HASP and SHERP replacement constructs integrated into the former L. (L.) major cDNA16 locus in the different L. (L.) major mutant lines generated for this study. Refer to S1 Table, Figs 1A and S1 for more details on the replacement constructs. (TIF) [file ppat.1006130.s002.tif]

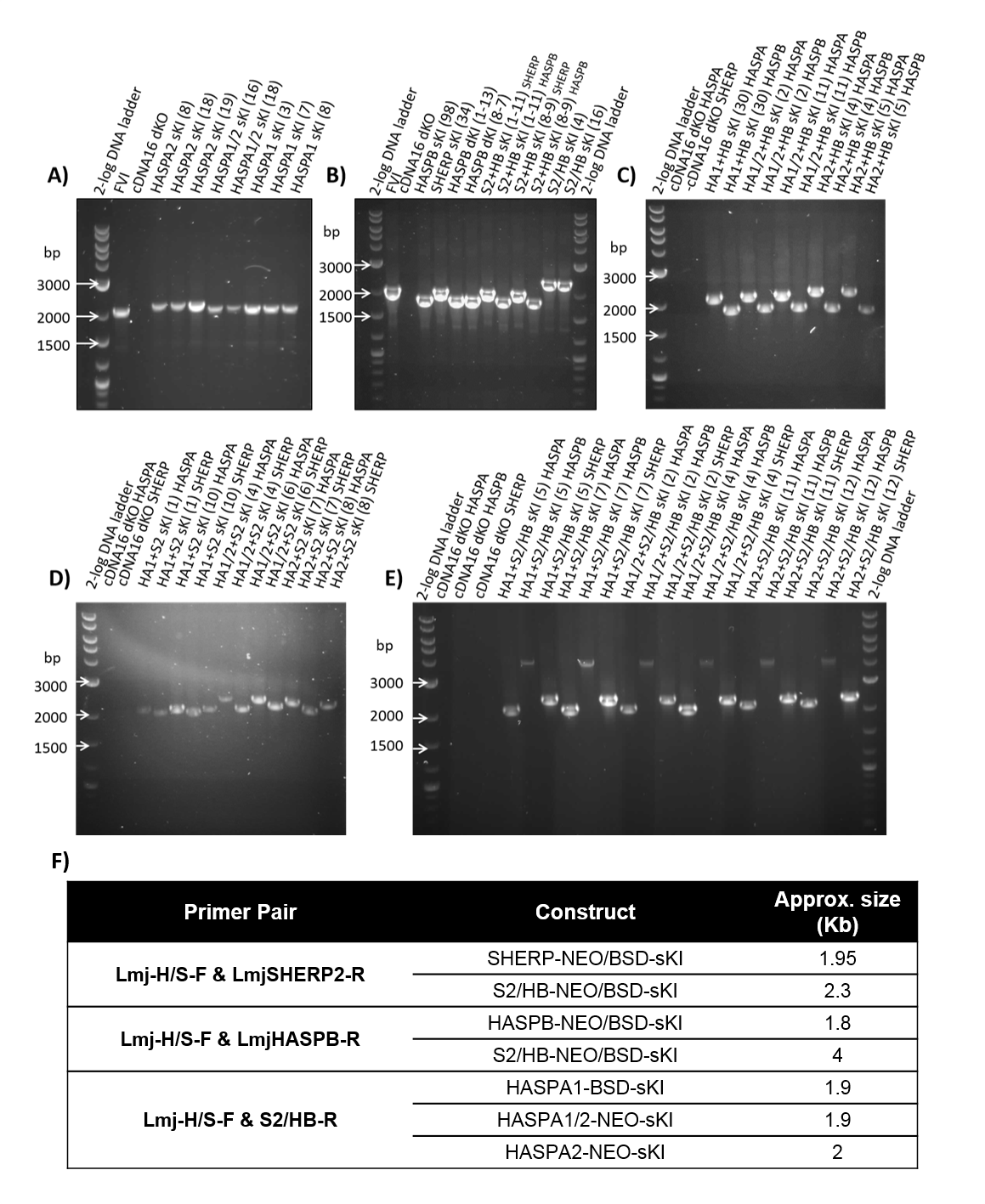

Supplement: S3 Fig — PCR screening of transfected L. (L.) major mutant clones for construct integration allowed rapid large scale selection of clones for further analysis (A-E). A minimum of 20 clones, where available, were first screened in this way. Gel images are representative for clones analysed in this study only, each identified by (number of clones) in the construct name. The gene names following the (number of clones) in images (B-E) refer to the gene targeted in that lane. (F) This Table lists the expected sizes of PCR products generated with the named primer pairs and targeted constructs. (TIF) [file ppat.1006130.s003.tif]

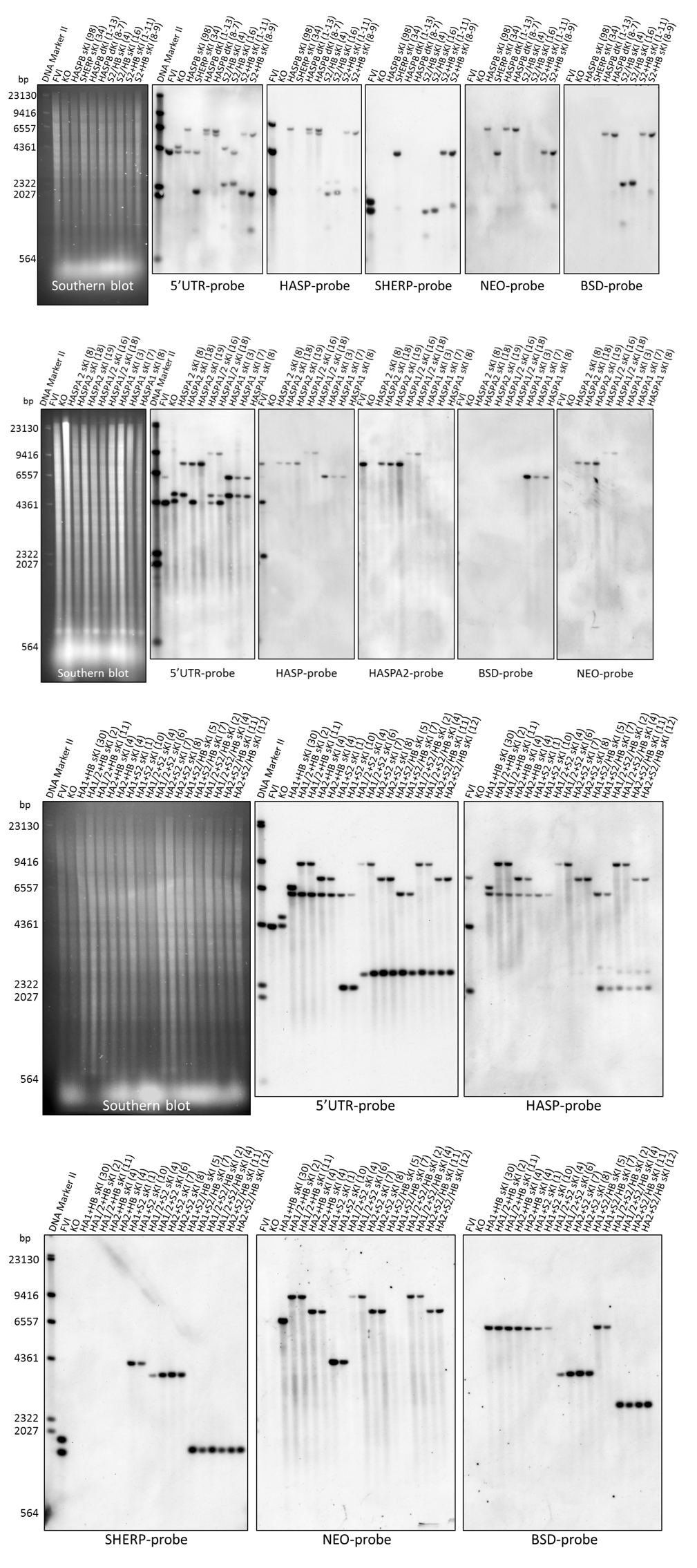

Supplement: S4 Fig — Southern blotting was used to confirm correct integration of replacement constructs and to exclude clones containing episomal constructs. Blots were probed multiple times with different DIG-labelled probes for the construct genes, resistance markers and the 5’ flanking region required for integration. Varying numbers of clones per mutant line were screened for clone selection. The blots shown are representative of the clones presented in this study only; the blot in Fig 1B was derived from these data. (TIF) [file ppat.1006130.s004.tif]

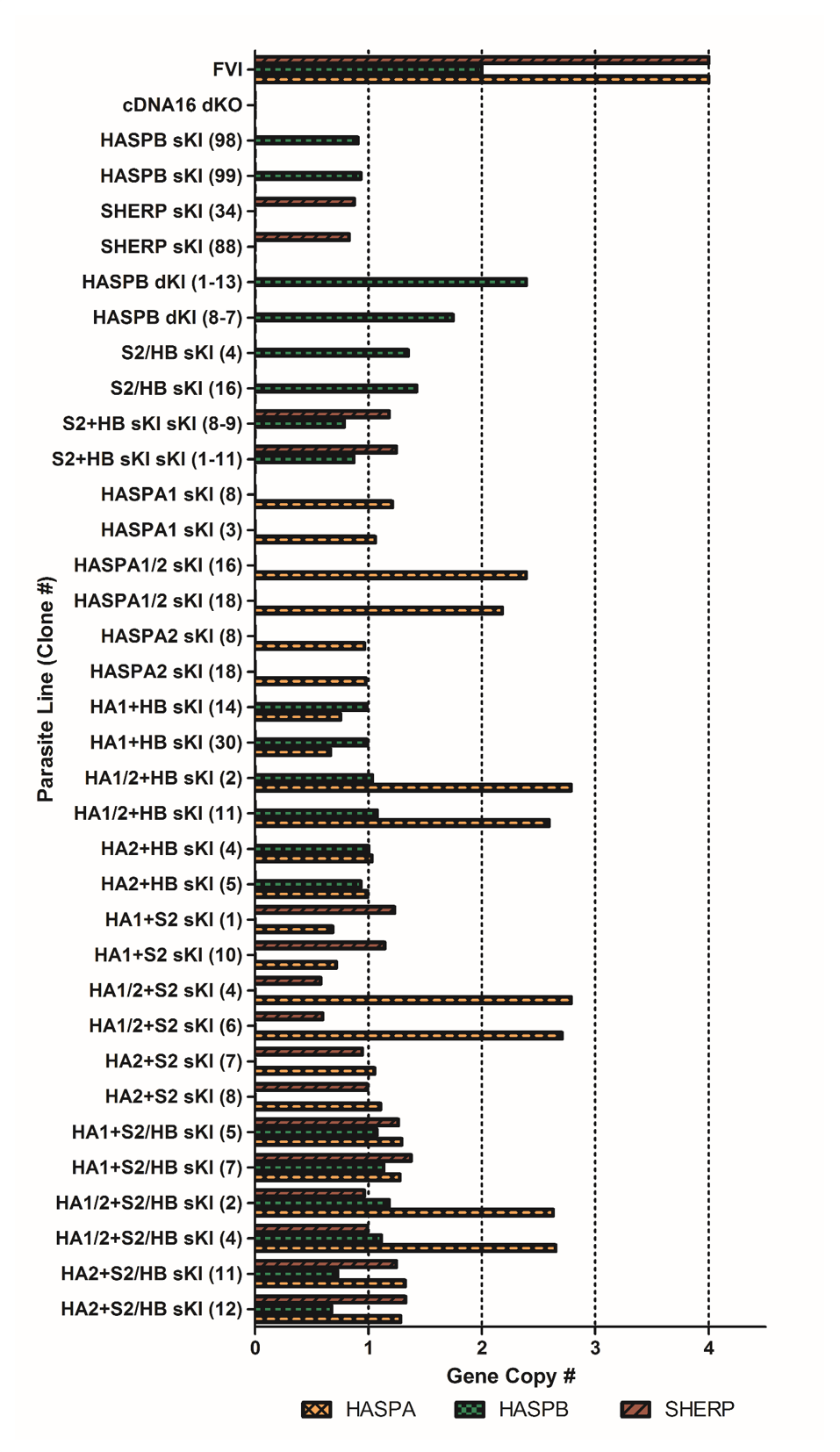

Supplement: S5 Fig — This expanded analysis of Fig 1C shows multiple clones per mutant line analysed for replacement construct copy number after transfection. All results were normalized internally against the Na/H antiporter-like protein on chromosome 23 and against FVI. (TIF) [file ppat.1006130.s005.tif]

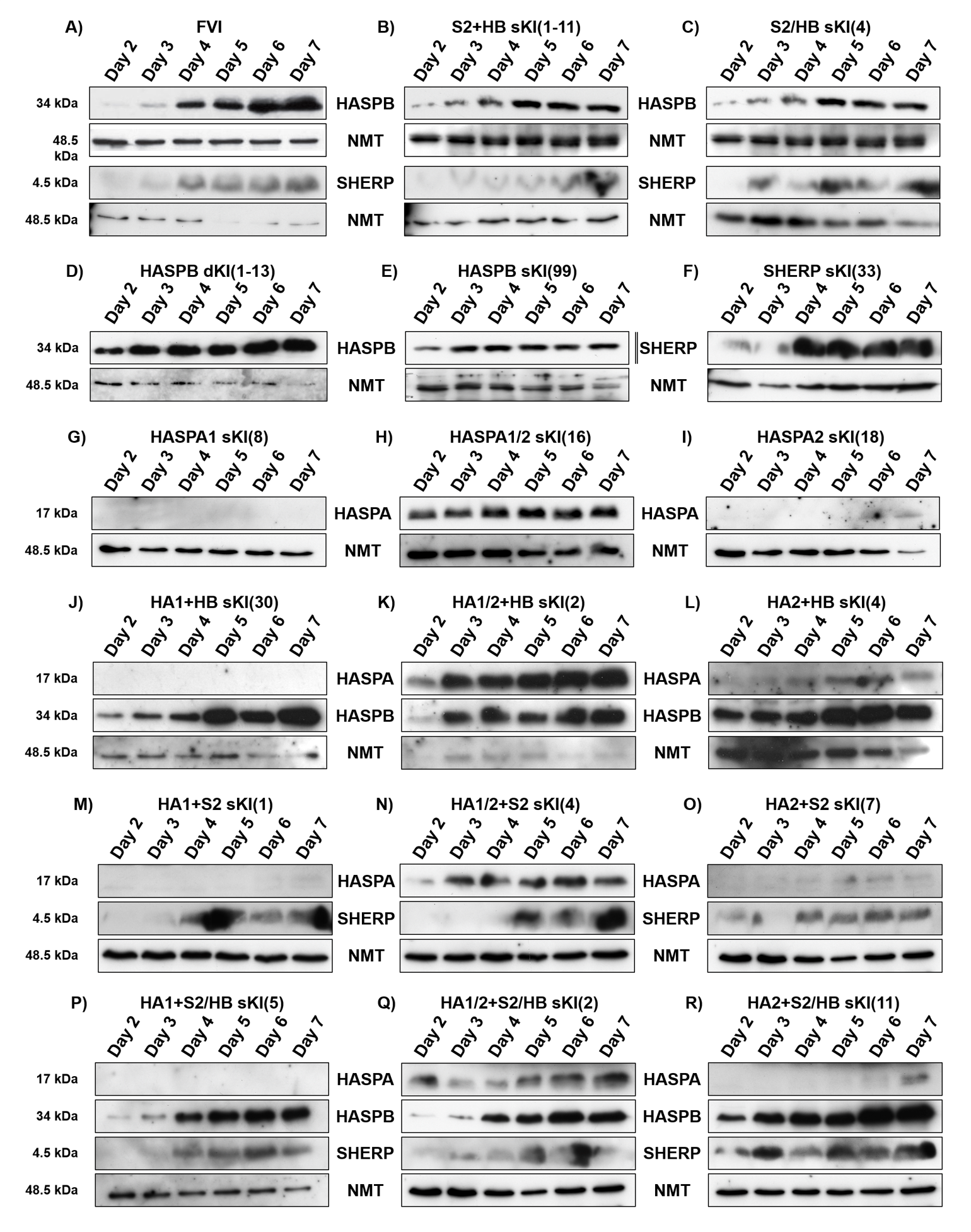

Supplement: S6 Fig — This expanded analysis of Fig 1D shows a single representative clone for each mutant line (labelled A-R) tested in this study. Clone identifiers are shown in brackets. For mutant lines shown in Fig 1D the alternative clone is shown here. (TIF) [file ppat.1006130.s006.tif]

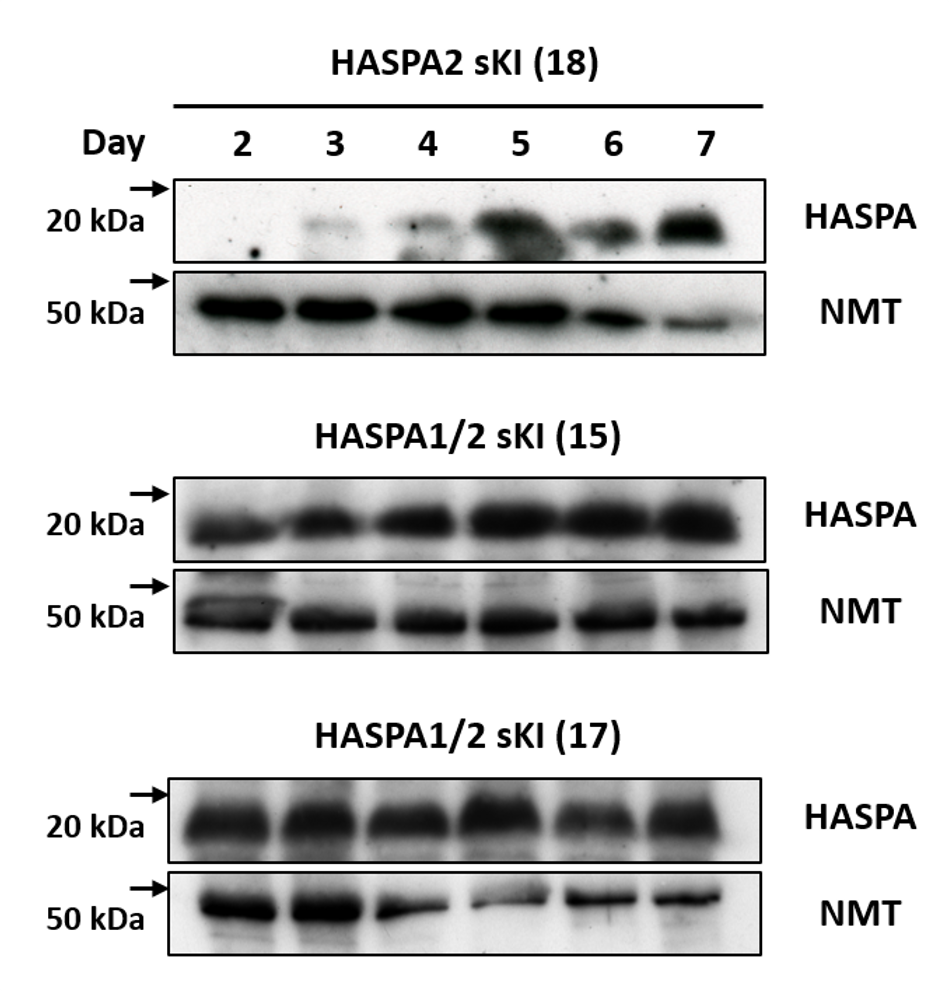

Supplement: S7 Fig — Immunoblots of one further HASPA2 sKI (18) and two further HASPA1/2 sKI (16 & 18) clones are shown. The HASPA2 sKI clone shows a similar HASPA expression pattern in promastigotes as observed in FVI and the HASPA2 sKI clone in Fig 1D. The additional HASPA1/2 sKI clones are expressing high and unregulated levels of HASPA as the clone shown in Fig 1D. The HASPA1/2 construct contains the same DNA fragments as the HASPA1 and HASPA2 constructs, which did not show the same level of expression. Thus the strong HASPA expression in the HASPA1/2 sKI line is not a clonal artefact, but a conserved property of the mutant line. (TIF) [file ppat.1006130.s007.tif]

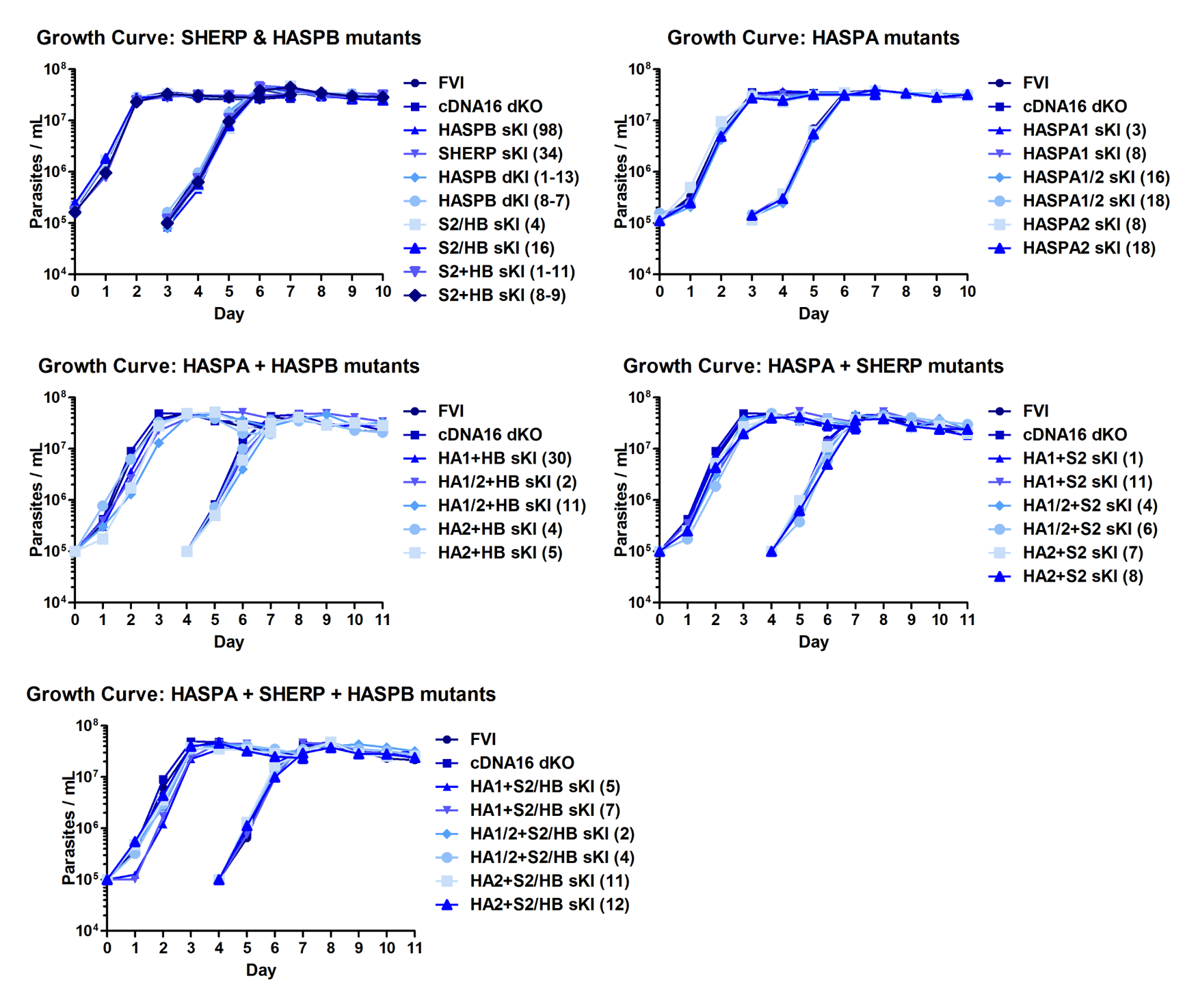

Supplement: S8 Fig — This expanded analysis of Fig 1E shows growth kinetics of selected clones of the different mutant lines. All clones were inoculated at 105 parasites/ml into 10 ml culture medium 199 and grown at 26°C for 7 days. Parasite numbers were counted once a day on a haemocytometer. These growth assays show that genetic transfection had no adverse effect on the viability and proliferation capacity of L. (L.) major in vitro. (TIF) [file ppat.1006130.s008.tif]

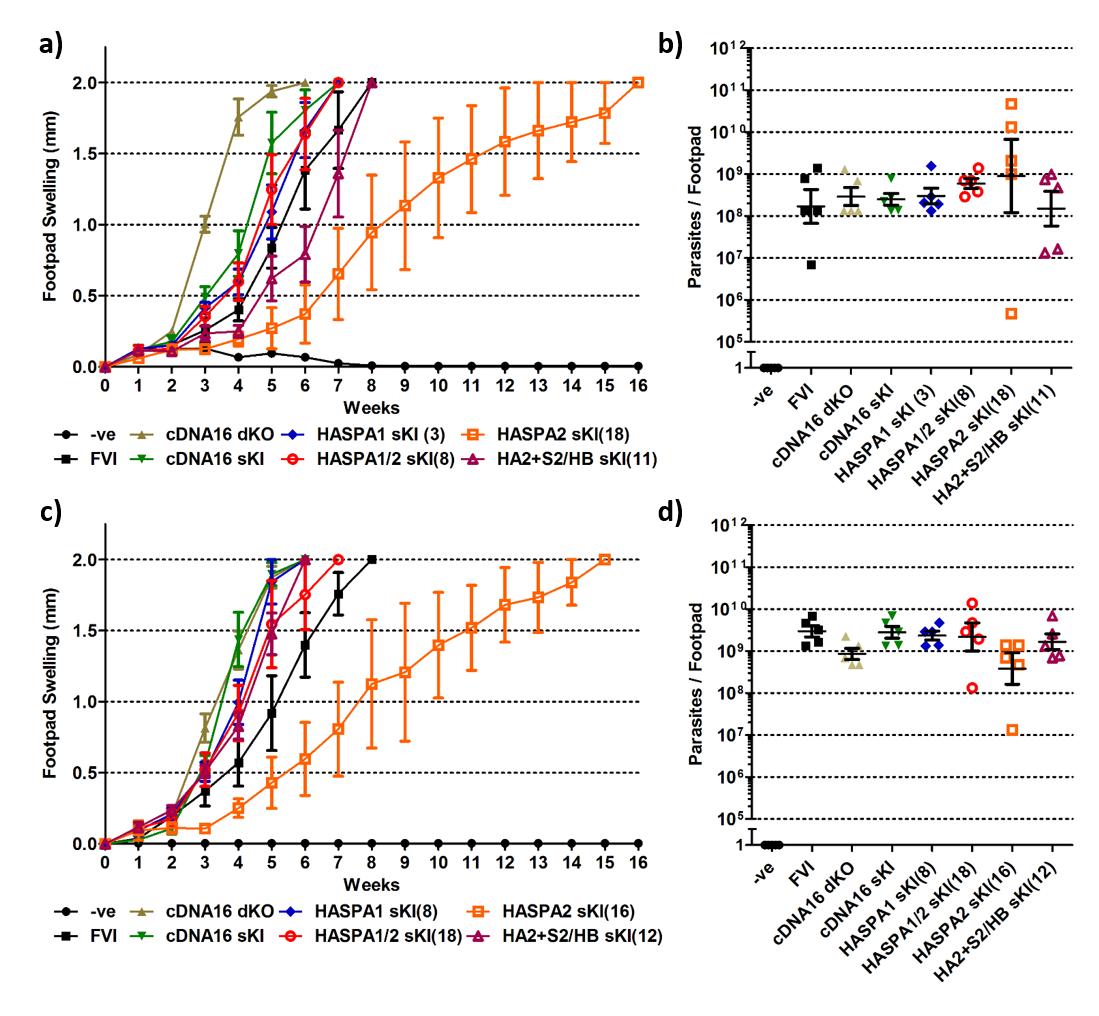

Supplement: S9 Fig — This figure shows the data presented in Fig 2C and 2D separated into the respective clones tested. a) and c) show the infection assay data by clone per line. Statistically significant differences (P<0.001) by repeat Friedman test measurements are observed for both HASPA2 sKI clones compared to all other lines/clones within the respective repeats. b) and d) show the LDA data by clone per line. b) represents the same clones as in a) and d) shows the same clones as in c). No statistically significant differences by Kruskal-Wallis test are observed for both HASPA2 sKI clones compared to all other lines/clones within the respective repeats. The clones shown in Figures a) and b) are HASPA1 sKI (3), HASPA2 sKI (18), HASPA1/2 sKI (8), HA2+S2/HB sKI (11). The clones shown in Figures c) and d) are HASPA1 sKI (8), HASPA2 sKI (16), HASPA1/2 sKI (18), HA2+S2/HB sKI (12). (TIF) [file ppat.1006130.s009.tif]

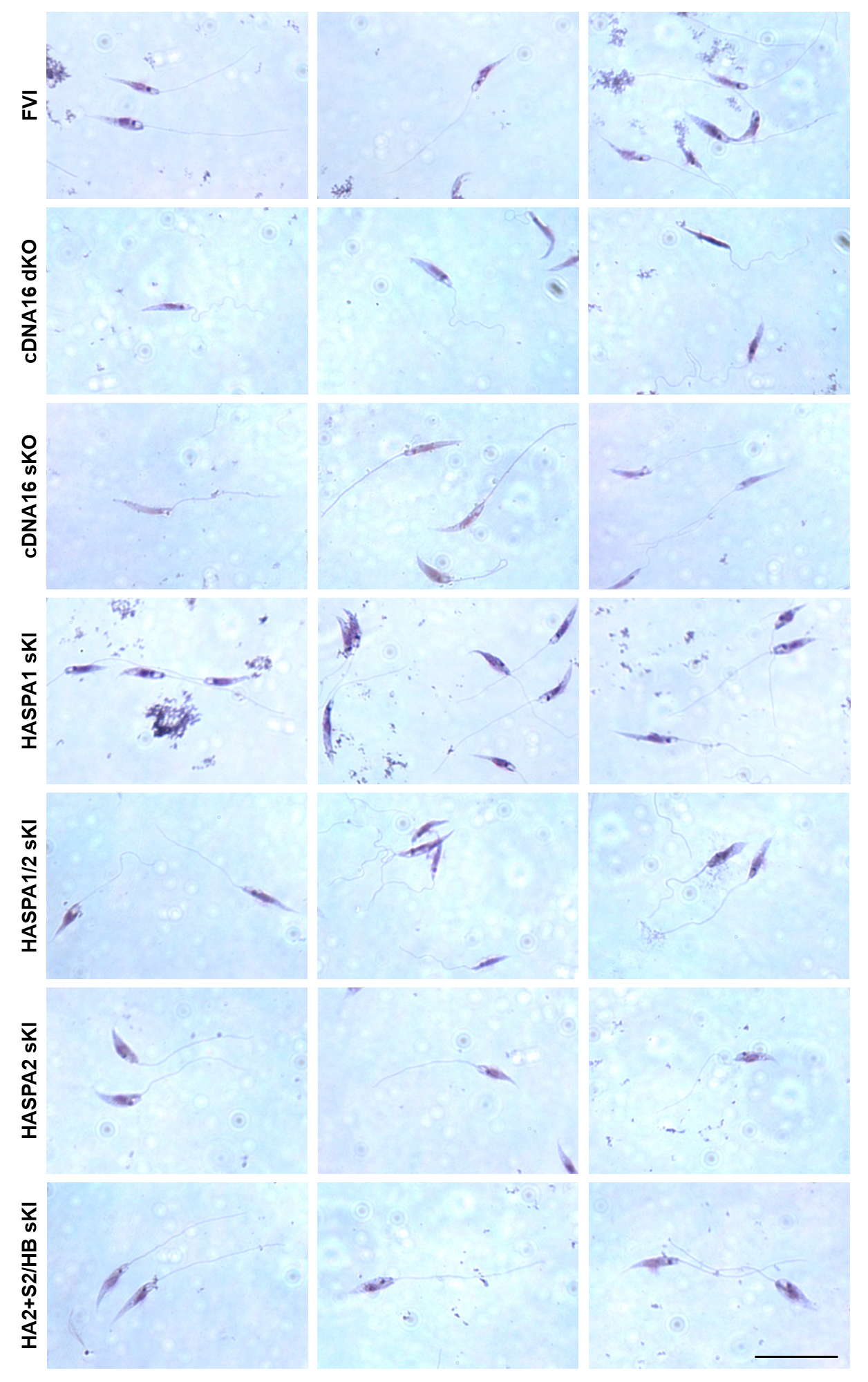

Supplement: S10 Fig — Three separate images are shown for each L. (L.) major line tested in three repeat agglutination assay experiments (refer to Fig 2B), showing Giemsa stained culture-derived metacyclics for subsequent morphometric verification. Size bar is equivalent to 10 μm. (TIF) [file ppat.1006130.s010.tif]

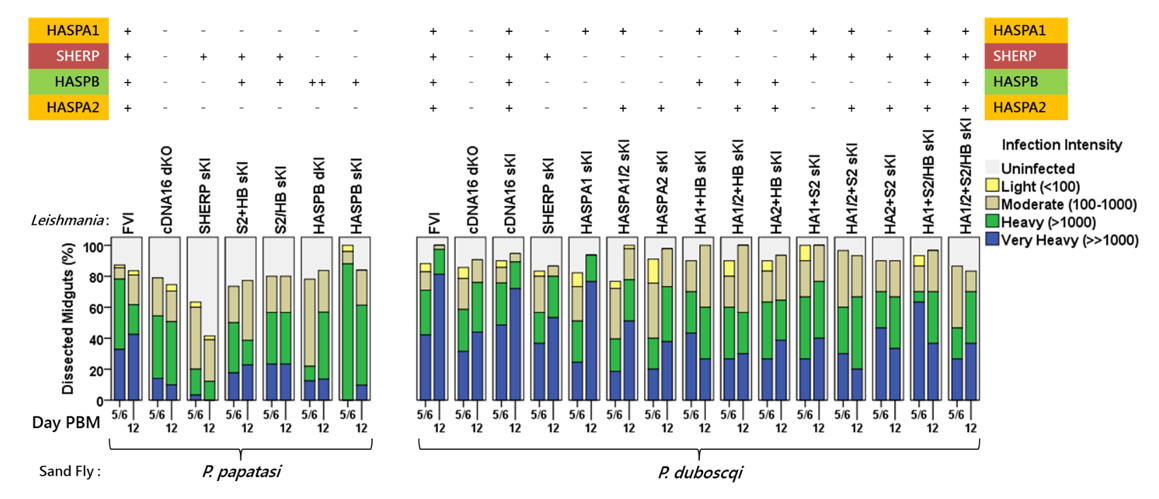

Supplement: S11 Fig — This expanded analysis of Fig 3A shows a single representative clone for all L. (L.) major mutant lines tested in sand flies. (TIF) [file ppat.1006130.s011.tif]

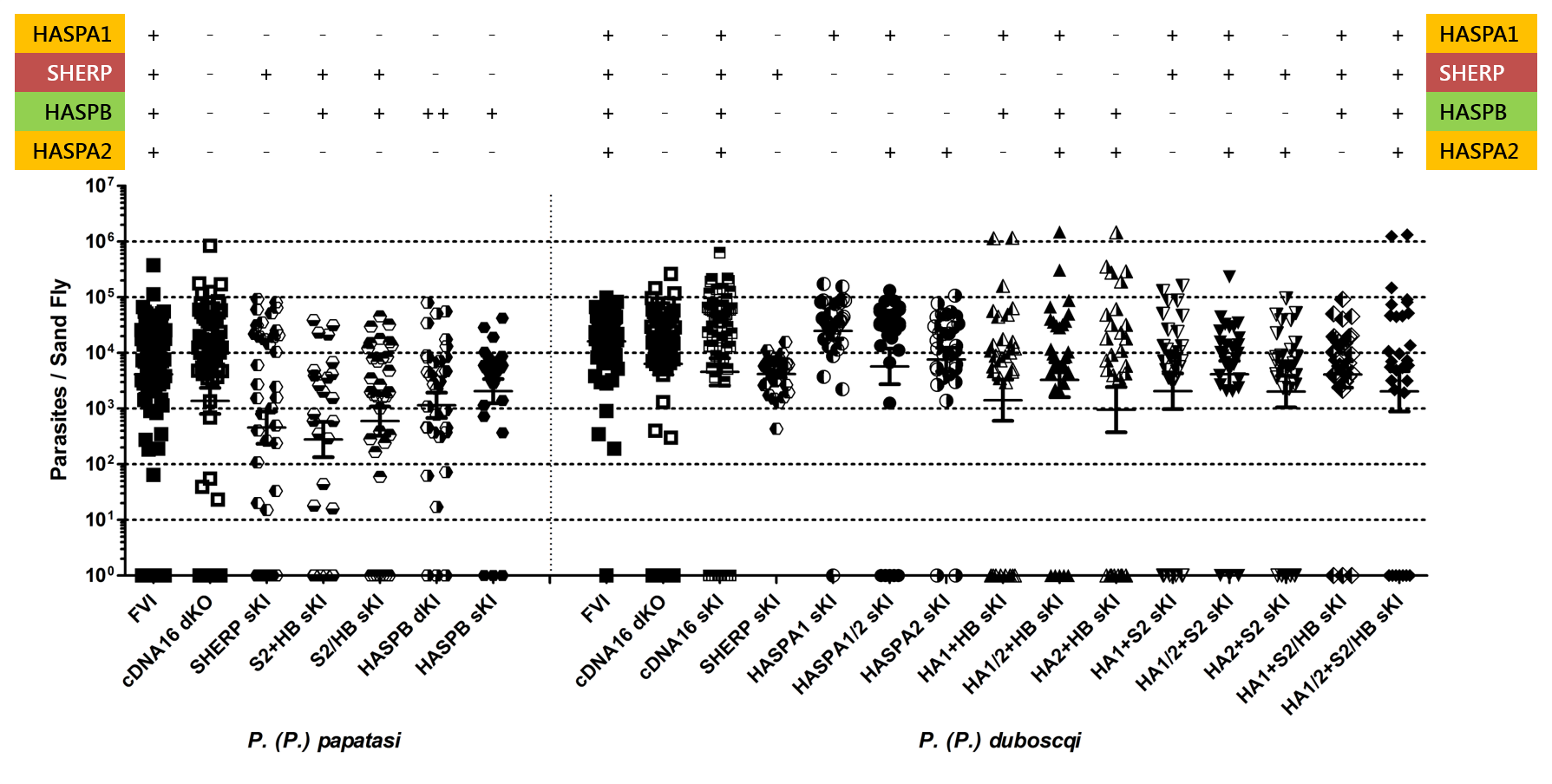

Supplement: S12 Fig — This expanded analysis of Fig 3B shows a single representative clone for all L. (L.) major mutant lines tested in sand flies. (TIF) [file ppat.1006130.s012.tif]

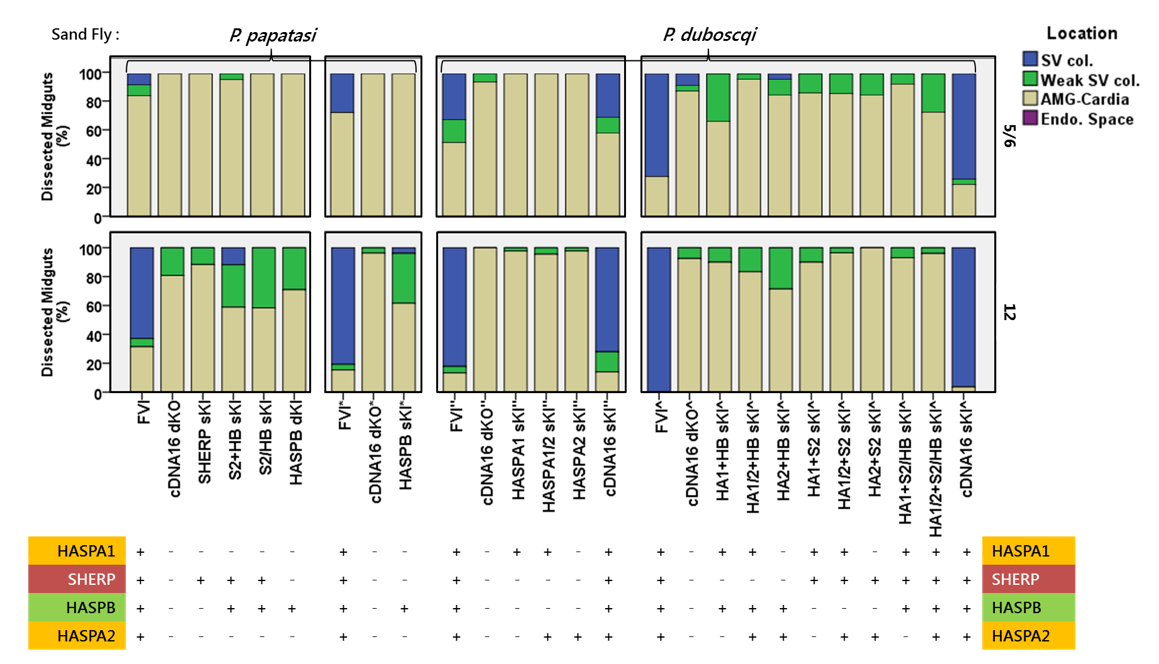

Supplement: S13 Fig — This expanded analysis of Fig 4 shows a single representative clone for all L. (L.) major mutant lines tested in sand flies. The *, “and ^ following the line names identify the separate sets of triplicate repeat experiments. (TIF) [file ppat.1006130.s013.tif]

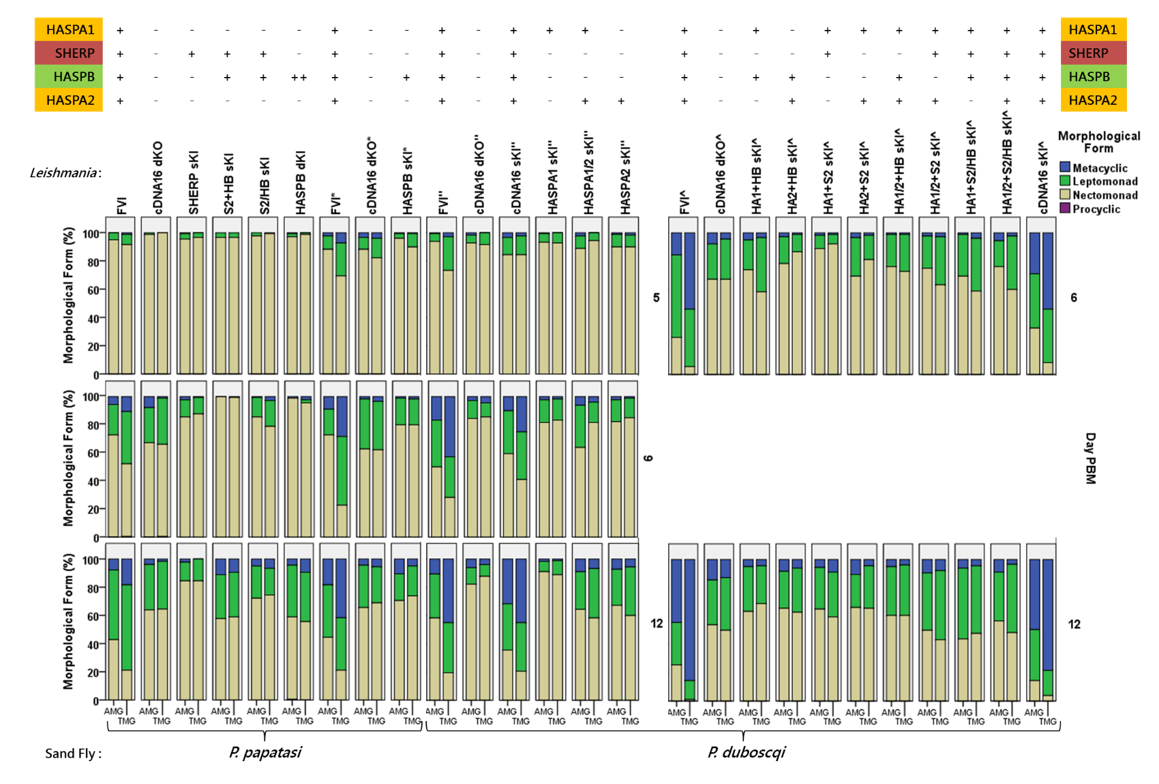

Supplement: S14 Fig — This expanded analysis of Fig 6 shows a single representative clone for all L. (L.) major mutant lines tested in sand flies. The *, “and ^ following the line names identify the separate sets of triplicate repeat experiments. (TIF) [file ppat.1006130.s014.tif]

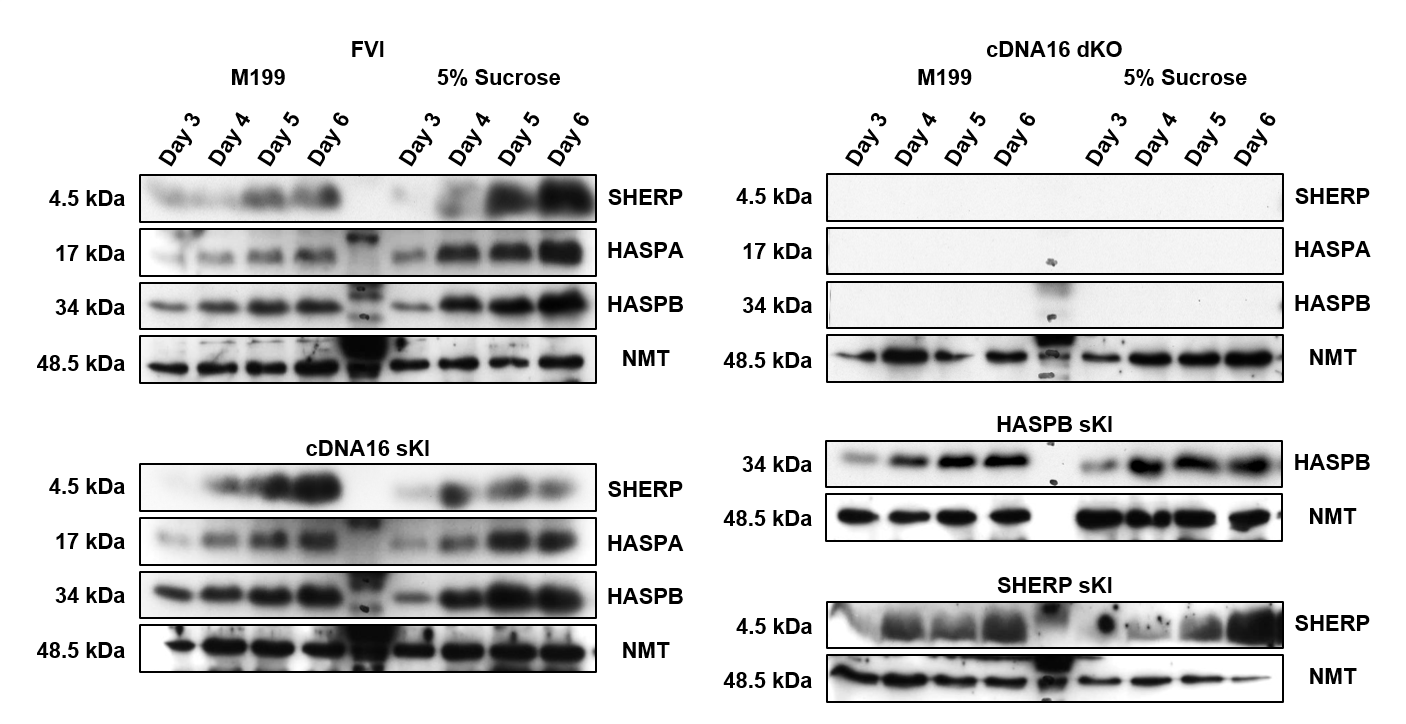

Supplement: S15 Fig — Immunoblot time course analyses of HASP and/or SHERP probed parasites (FVI, cDNA16 dKO, cDNA16 sKI, HASPB sKI and SHERP sKI) differentiated either in M199 or in 5% sucrose/PBS until day 7 p.i. The latter resembles more closely the nutrient depleted conditions during parasite differentiation in the sand fly midgut following blood meal defecation; parasites were transferred from M199 into 5% sucrose/PBS at day 3 p.i. (TIF) [file ppat.1006130.s015.tif]

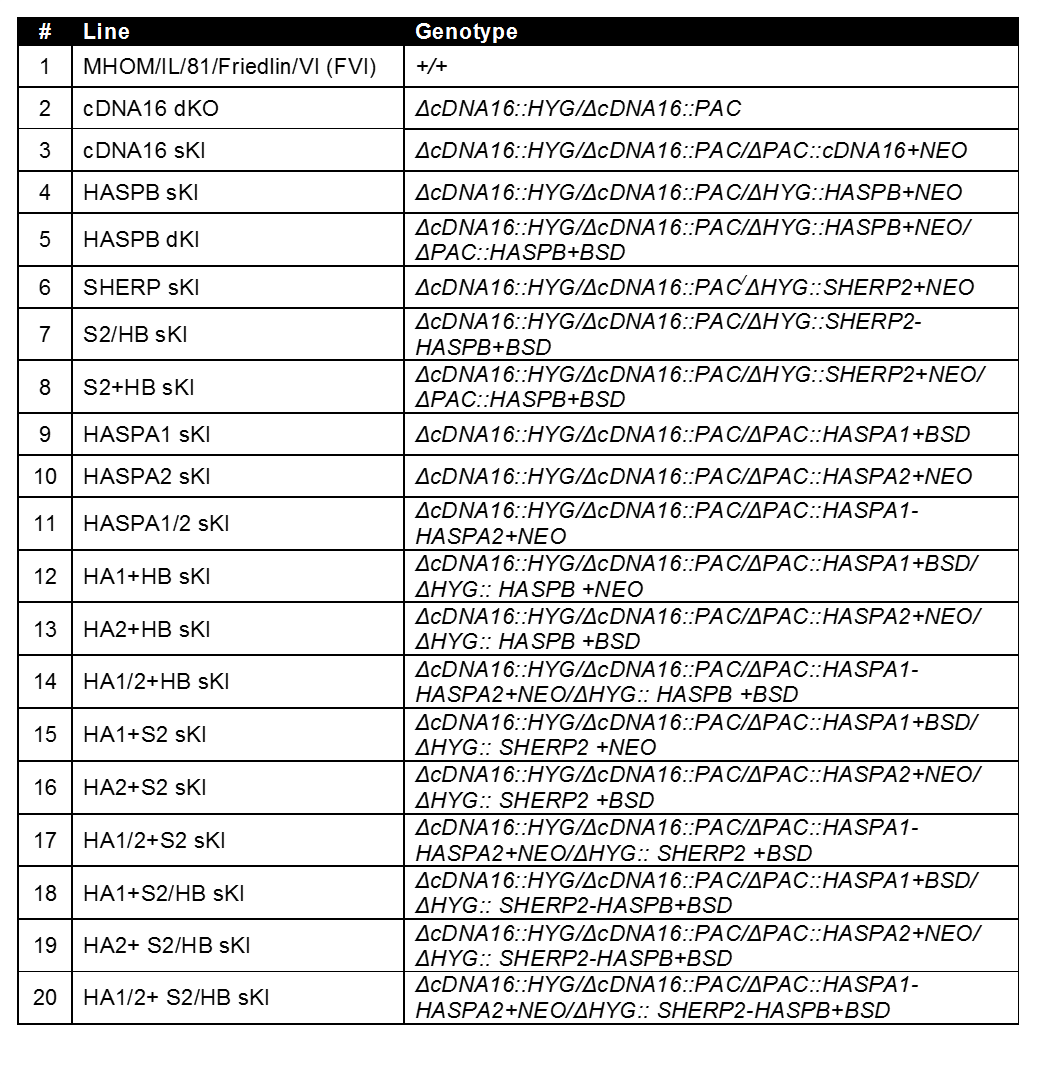

Supplement: S1 Table — This expanded version of Table 1 shows all L. (L.) major lines used in this study and included in the supplementary figures. (TIF) [file ppat.1006130.s016.tif]

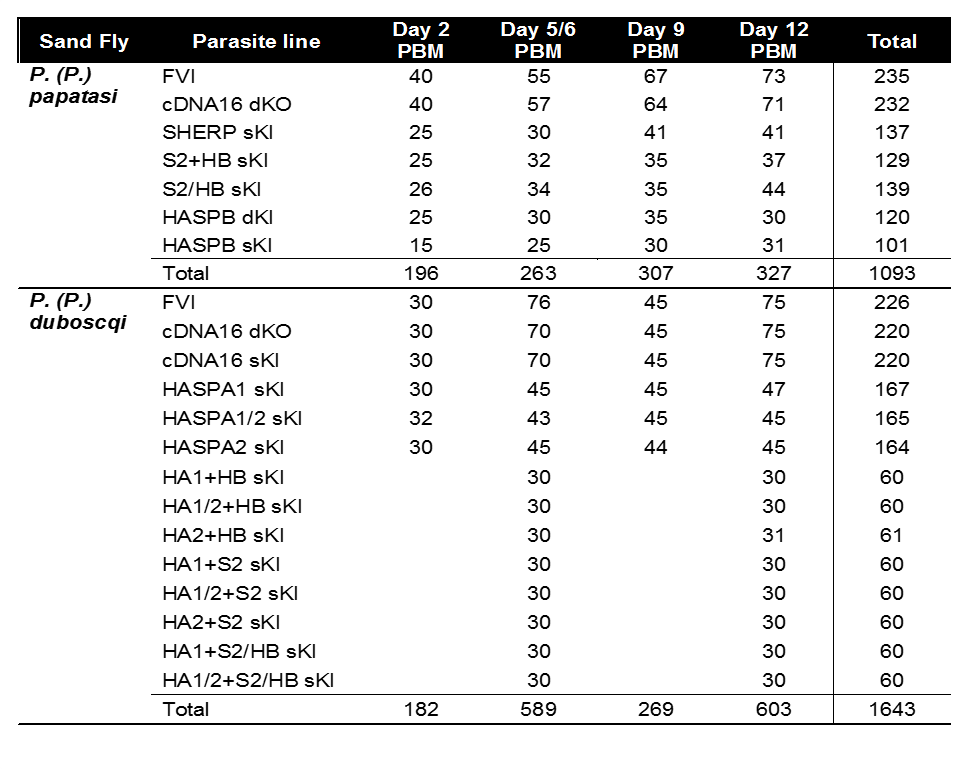

Supplement: S2 Table — The table lists the total numbers of female sand flies dissected per line per day PBM; see Figs 3A and 4. (TIF) [file ppat.1006130.s017.tif]

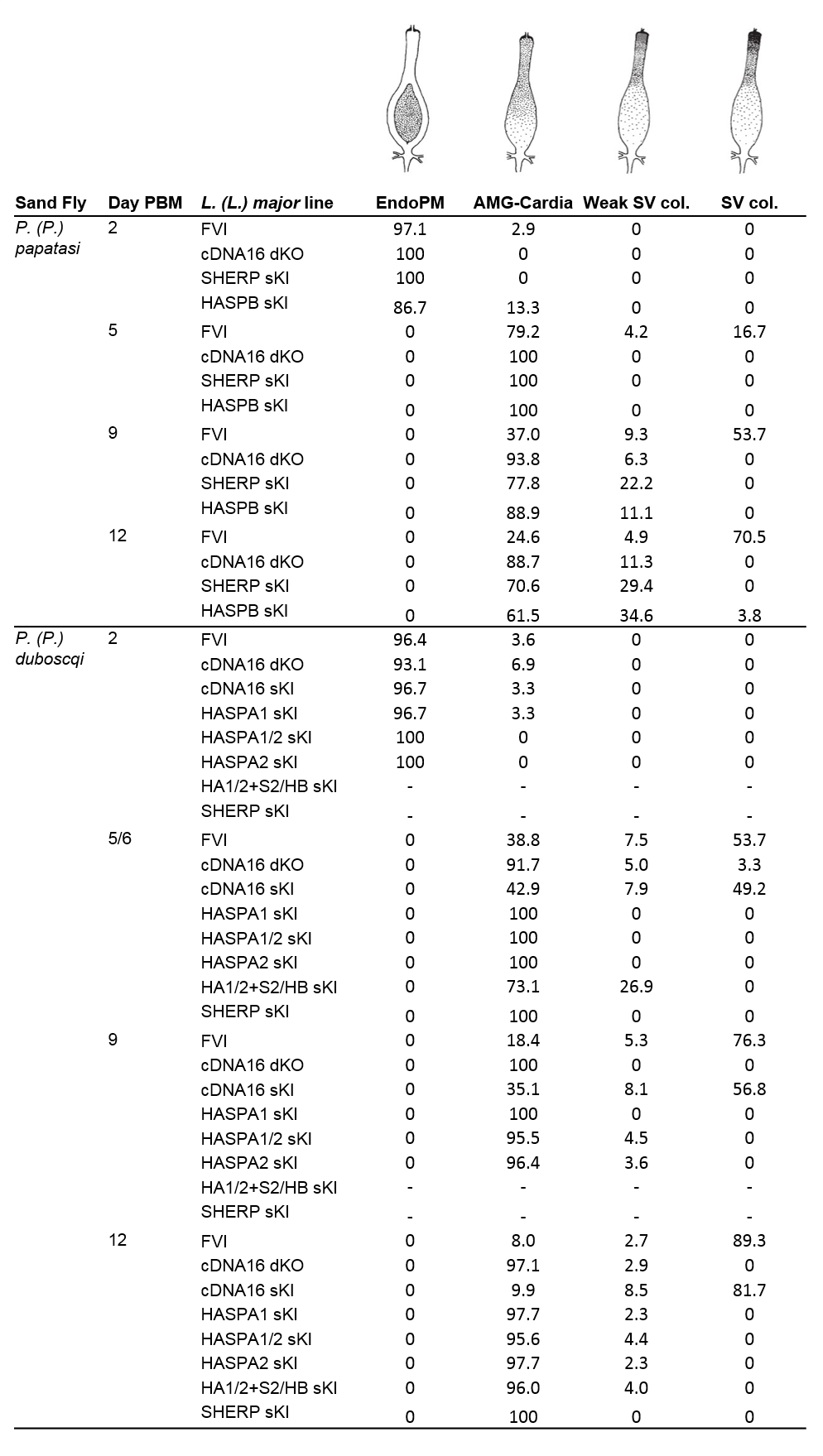

Supplement: S3 Table — Fig 4 results are presented as percentages of all sand flies analysed per mutant line. Refer to S1 Table for information on dissected sand fly numbers per respective L. (L.) major line tested. (TIF) [file ppat.1006130.s018.tif]

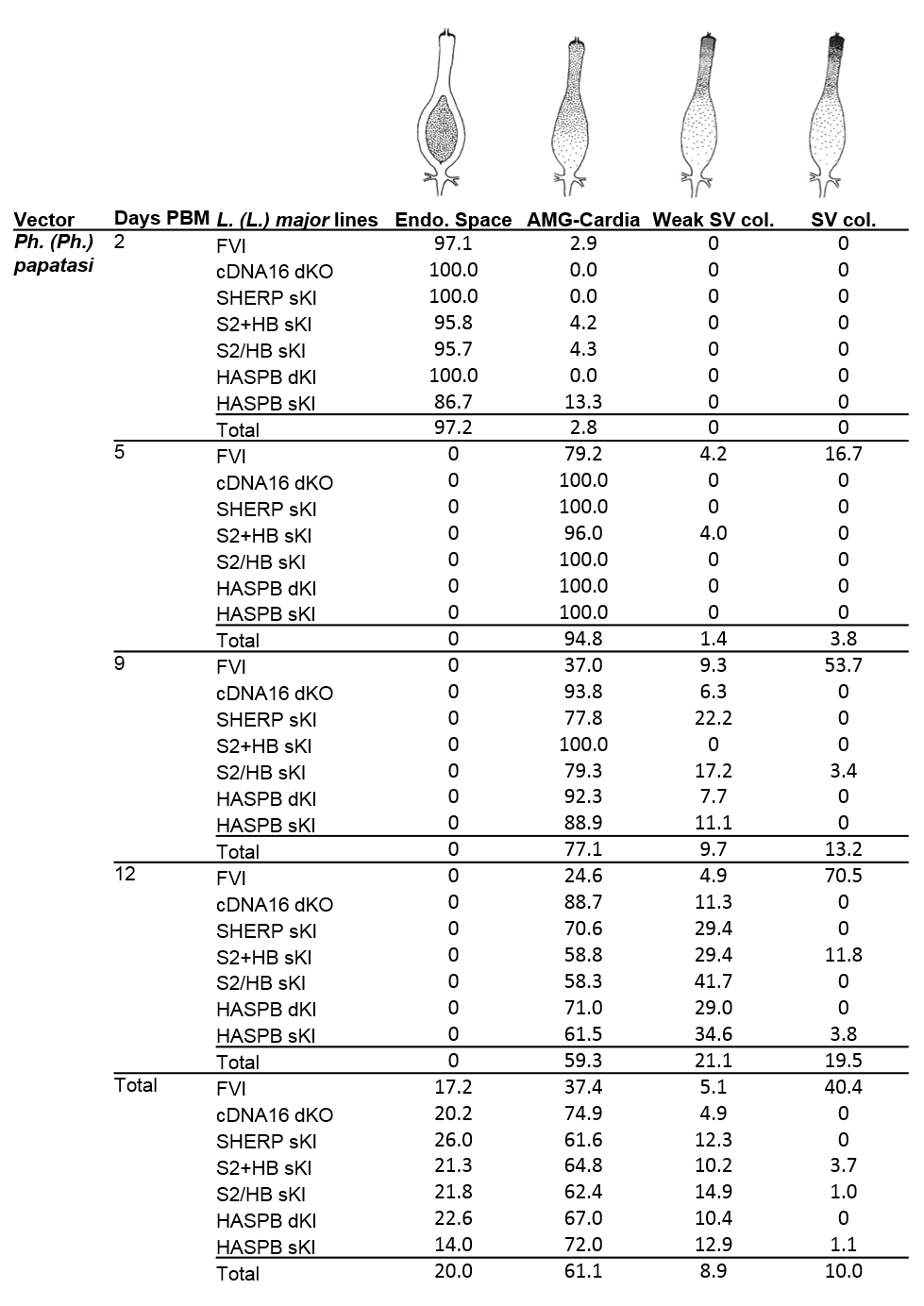

Supplement: S4 Table — This expanded version of S3 Table shows all the L. (L.) major mutant lines infected into P. papatasi. (TIF) [file ppat.1006130.s019.tif]

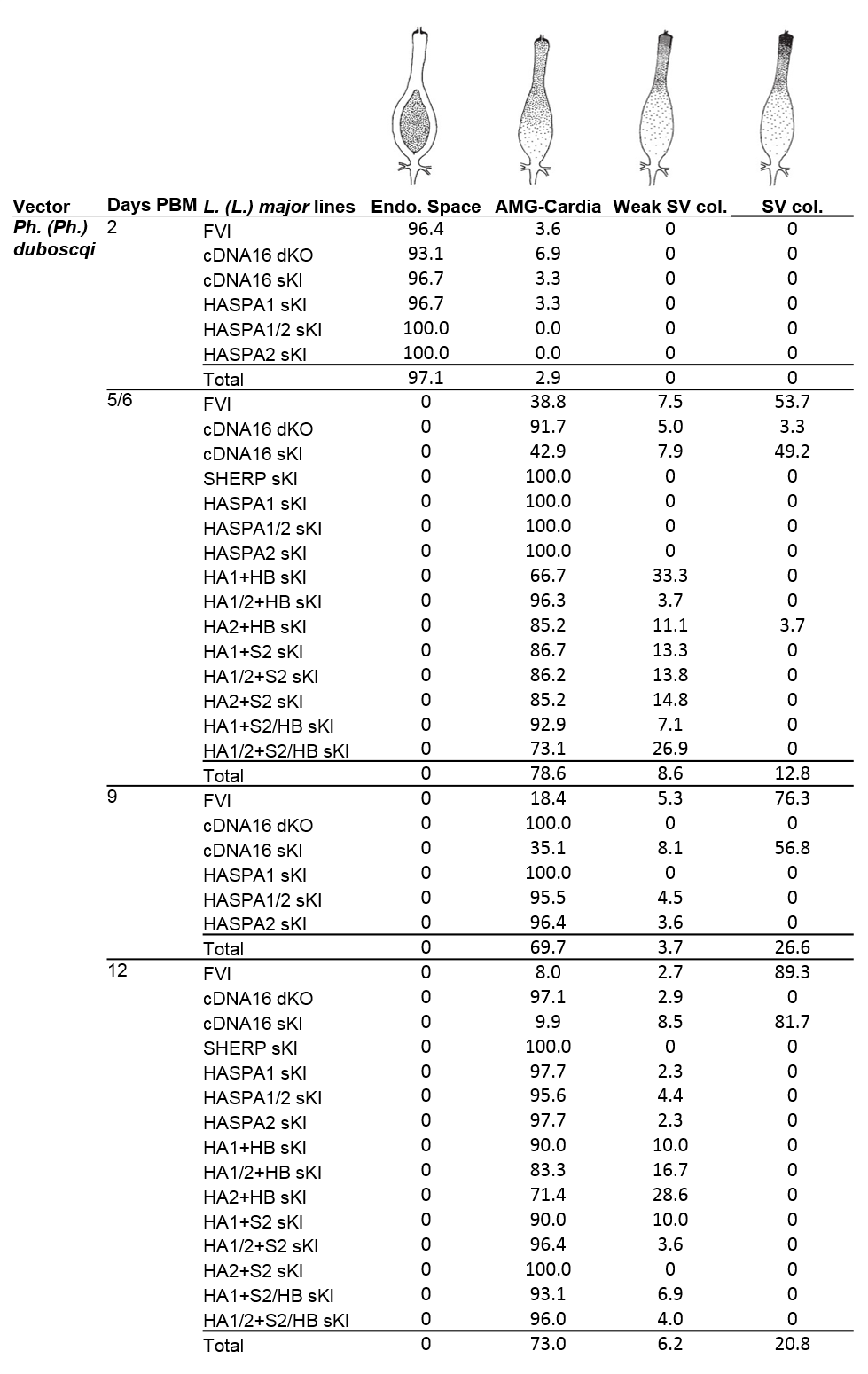

Supplement: S5 Table — This expanded version of S3 Table shows all the L. (L.) major mutant lines infected into P. duboscqi. (TIF) [file ppat.1006130.s020.tif]

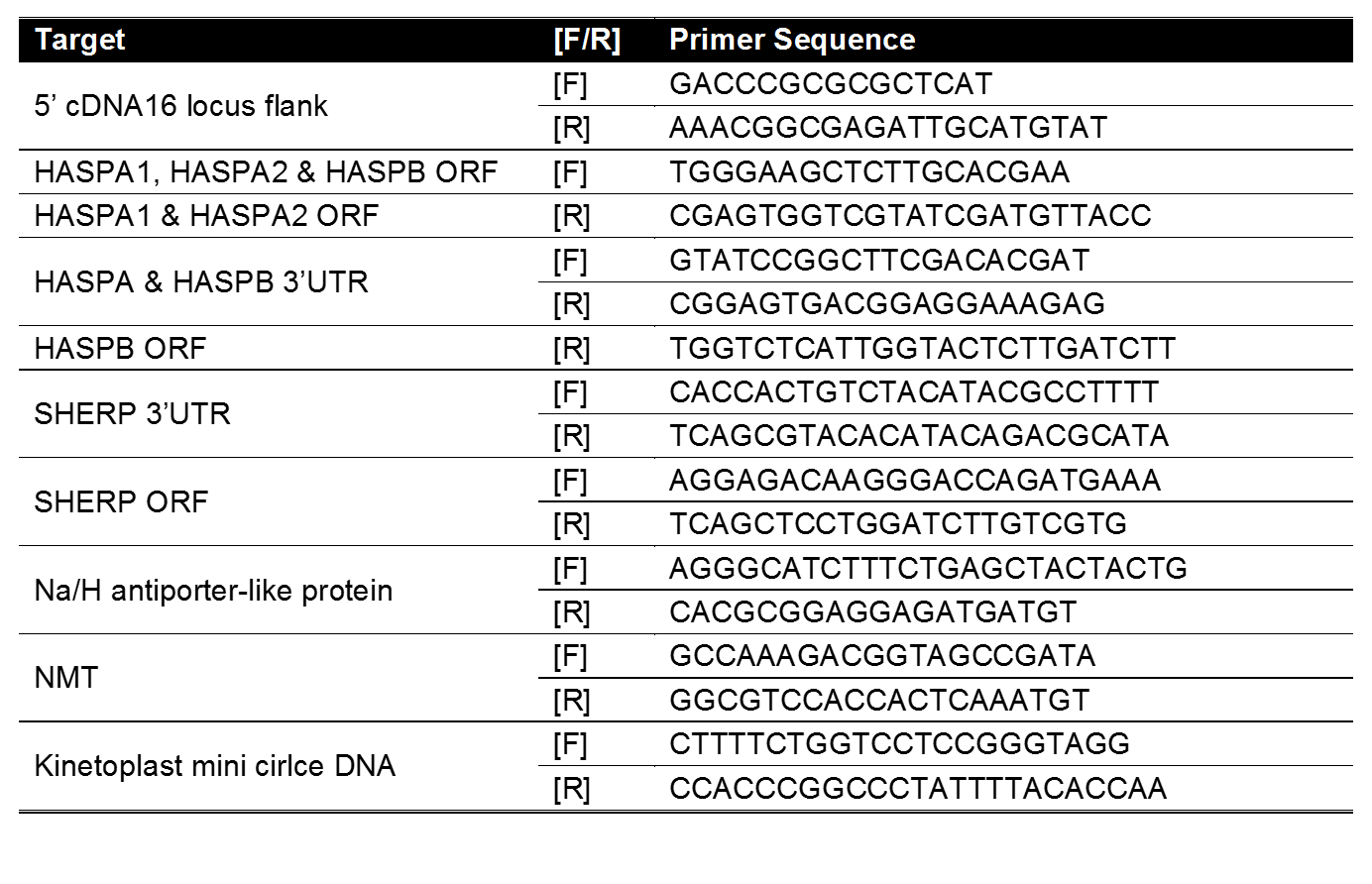

Supplement: S6 Table — (TIF) [file ppat.1006130.s021.tif]
